# Fatigue in patients with syndromic heritable thoracic aortic disease: a systematic review of the literature and a qualitative study of patients’ experiences and perceptions

**DOI:** 10.1186/s13023-023-02709-2

**Published:** 2023-05-19

**Authors:** Gry Velvin, Heidi Johansen, Amy Østertun-Geirdal, Trine Bathen

**Affiliations:** 1 TRS National Resource Centre for Rare Disorders, Sunnaas Rehabilitation Hospital, 1450 Nesoddtangen, Norway; 2 Department of Social Work, Child Welfare and Social Policy, Faculty of Social Science, Oslo Metropolitan University, Oslo, Norway

**Keywords:** Fatigue, Exhaustion, Heritable thoracic aortic disease, Marfan syndrome, Loeys-Dietz syndrome and vascular Ehlers-Danlos syndrome, Systematic review, Qualitative focus groups

## Abstract

**Introduction:**

The purpose of this study was to explore the literature on fatigue in patients with syndromic heritable thoracic aortic disease (sHTAD), including Marfan syndrome (MFS), Loeys-Dietz syndrome (LDS), vascular Ehlers Danlos syndrome (vEDS) and other sHTADs, critically appraise and synthesize the relevant literature. We also aimed to investigate how adults with sHTAD experience and perceive fatigue, and to discuss clinical implications and direction for further research.

**Methods:**

First, a systematic review was performed by searching the published literature in all relevant databases and other sources until 20th October 2022. Second, a qualitative focus group interview study was conducted of 36 adults with sHTADs (LDS n = 11, MFS n = 14, vEDS n = 11).

**Results:**

In the systematic review, 33 articles satisfied the eligibility criteria (3 reviews and 30 primary studies). Of the primary studies: 25 dealt with adults (MFS n = 17, MFS/EDS n = 1, EDS n = 2, LDS/vEDS n = 3, different sHTADs n = 2), 5 with children (MFS n = 4, different sHTADs n = 1). Twenty-two were cross-sectional quantitative studies, 4 prospective and 4 qualitative studies. The quality of the included studies was mostly good, but many had small sample sizes, low response rates and/or participants without verified diagnosis. Despite these limitations, studies indicated high prevalence of fatigue (ranging from 37 to 89%), and fatigue was associated with both health and psychosocial aspects. Few studies found that fatigue was associated with disease-related symptoms. In the qualitative focus groups most of the participants reported that they had experienced fatigue which influenced several aspects of life. Four themes related to fatigue were elucidated: (1) different diagnoses–different fatigue?, (2) the nature of fatigue, (3) searches for causes of fatigue, (4) dealing with fatigue in daily life. The four themes seemed mutually interrelated in terms of barriers, strategies and facilitators for dealing with fatigue. The participants experienced fatigue as a consistent dilemma between self-assertion and inadequacy. Fatigue seems to influence several aspects of daily life and may be one of the most debilitating symptoms of having a sHTAD.

**Conclusion:**

Fatigue seems to negatively impact the lives of people with sHTADs and should be recognized as an important aspect in the lifelong follow-up of these patients. The life-threatening complications of sHTADs may result in emotional stress, including fatigue and the risk of developing a sedentary lifestyle. Research and clinical initiatives should consider rehabilitation interventions aiming at postponing the onset or reducing symptoms of fatigue.

**Supplementary Information:**

The online version contains supplementary material available at 10.1186/s13023-023-02709-2.

## Introduction

### Heritable thoracic aortic disease

Heritable thoracic aortic disease (HTAD) is a term that defines a large group of disorder characterized by the occurrence of aortic events, mainly represented by aneurysm or dissection [1]. HTAD can be classified as non-syndromic if the disorder is limited to the aorta, and syndromic when associated to extra aortic features [1–3]. Genetic testing is essential since it allows confirmation of the etiological diagnoses for HTAD. An extensive list of human genes and other clinical features associated with HTADs is mentioned in several papers [4–6]. Renard et al. [6] found that approximately 53 candidate genes were associated with HTADs, but only 11 genes (*COL3A1, FBN1, SMAD3, TGFB2, TGFBR1, TGFBR2, ACTA2, MYH11*, *MYLK, LOX and PRKG1*) were identified as “HTAD” genes as they were assessed as having a “definitive” and “strong” gene-disease association during the curation process [6]. Mutations in the five last genes described above are known as non-syndromic HTAD (nsHTAD), as they are associated with vascular manifestation alone [2, 6, 7]. Mutations in the six first genes are known to cause syndromic HTAD (sHTAD), with systemic manifestations and genetic phenotype, including cardiovascular, musculoskeletal, craniofacial and ocular systems, and cutaneous features [4, 5]. The most common diseases of sHTAD are Marfan syndrome (MFS), Loeys-Dietz syndrome (LDS) and vascular Ehlers-Danlos syndrome (vEDS) [2, 4, 7]. The focus of the present study is sHTAD.

The most serious complications in sHTADs are related to the risk of aneurysm and dissection of aorta and other large arteries [2]. Life-threatening complications can require emergency intervention, with increased risk of morbidity and mortality [2, 8]. Because of the risk of aortic dissection, many patients are advised to refrain from contact sport, to limit their physical exertion and to control their blood pressure strictly [9, 10]. Unfortunately, they often follow by a sedentary lifestyle [10–13]. Many have skeletal signs with hypermobile joints, chest deformities and scoliosis [4–6]. Physical impairment, chronic pain and fatigue associated with sHTADs may be exacerbated by the fact that most sHTADs have no effective treatment or cure [2, 4, 14]. Living with a sHTAD may be vastly more complex than just its medical features [15–18]. Many aspects of an individual`s life may be affected such as family life, education, work-life and leisure activities [13, 19, 20].

### Rationale for the study in the context of what is already known

In recent years, fatigue has increasingly been studied in genetic conditions, and recognized as an important clinical factor affecting several aspects of patients’ lives [21]. Through our work in a resource center for rare diseases, we frequently encounter patients who report fatigue as a serious problem. Many patients also experience lack of understanding and support from professionals regarding their fatigue. There is a risk that health professionals do not pay attention to fatigue because it is overshadowed by other more potential life-threatening aspects of the disease. Often, medical causes of fatigue may be ignored [21].

A challenge is that fatigue is a complex phenomenon lacking a clear definition. Many studies fail to explain their definition of fatigue [21, 22]. Authors often do not explicitly define fatigue, but rather imply its meaning by the concept associated with the instrument used for measuring fatigue. Other terms used in the literature for fatigue included “tiredness”, “exhaustion” or “lack of vitality” [23]. A common definition of fatigue is “an overwhelming sense of tiredness, lack of energy and feeling of exhaustion, mental, physical or both” [24]. Other attempts to classify fatigue according to the affected domains are (motor versus cognitive) or the presumptive origin (central versus peripheral) [25]. Penner et al. [25] have proposed a unifying taxonomy that discriminates between fatigue (in sense of self-perception) and performance (fatigability). Self-perceived fatigue can be quantified by scales that cover physical, psychosocial and/or cognitive aspects, whereas fatigability can be quantified by measuring the decline in performance of given tasks (such as motor fatigability and decline reaction time, often measured in laboratories) [22, 25]. In this study, the focus is on self-perceived fatigue.

There is a wealth of distinct and often discrepant scales that have been designed to measure both generic and disease-specific fatigue [21, 23]. To the best of our knowledge, no disease specific scales have been developed to measure self-perceived fatigue in persons with sHTADs. Brown et al. [26], Penner et al. [25] and McCabe et al. [27] have described vitality as the opposite of fatigue, with a low degree of vitality indicating severe fatigue. The RAND version of SF-36 Health Survey [28] is a health related quality of life measure where the subscale of vitality is defined as a scale for measuring general energy, lack of vitality, i.e. similar to fatigue [26, 27, 29] 36-Item Short Form Survey (SF-36) Scoring Instructions | RAND. Vitality is found to strongly correlate with different validated measures of fatigue [26, 27]. Vitality can also be used to assess a general level of fatigue in the preceding 4 weeks [29]. Overman et al. [29] indicate that the scores of 35 and lower of the SF-36 vitality score (0–100) indicate severe fatigue. We therefore included studies with outcomes on SF-36 vitality (SF-36vt) in the review part of this study.

In chronic diseases, fatigue may have multiple contributing factors e.g. sleep disorders, pain, reduced physical activity, depression and pharmacotherapy [25, 30]. As complex disorders, there are numerous factors in sHTADs that could interfere with physical, psychological and social function [31]. It is hypothesized that a number of factors may cause fatigue in patients with sHTADs such as physical (cardiovascular and respirators factors, working capacity, use of beta-blockers, reduced visual acuity and joint hypermobility), and mental/psychological comorbidity (cognitive dysfunction and psychological distress) [13, 16, 17, 19, 20, 31, 32]. The research on prevalence, associations, patient experiences and impact of fatigue in sHTADs appears to be fragmented and not well known. We therefore decided to undertake an overview of the literature and combine it with a qualitative study of the patients’ perceptions and coping strategies regarding self-perceived fatigue. We did this in the hope of developing a more evidence-based clinical practice.

### The aims of the study

1. To identify, critically appraise and synthesize available research about self-perceived fatigue in sHTADs.
2. To investigate the experiences and perceptions of fatigue in adults with different sHTADs.
3. To investigate key concepts of fatigue, identify knowledge gaps, and discuss clinical implications and direction for further research on fatigue in sHTADs.

## Methods and materials

### Systematic review

#### Study design

Owing to the limited number of studies on fatigue in sHTADs, all studies stating that at least one aim was to study fatigue in sHTADs were included in the review. The review was conducted according to the recommendation for systematic reviews [33, 34], and PRISMA checklist for systematic reviews [35] (Additional file 1). Each study was examined independently [33, 34, 36]. Standardized specific criteria were used to critically appraise the different types of studies [37–39]. In the evaluation of knowledge on fatigue, only the fatigue part of the studies was addressed. The review protocol is included in Additional file 2.

#### Search strategy

Systematic searches were conducted on relevant literature until October 2022, in PubMed, CINAHL, Embase, Ovid, MEDLINE, the Cochrane and Google Scholar. First, we conducted a search of terms related to HTAD, resulting in 15,872 hits. Then, we conducted a search of terms related to fatigue, resulting in 9547 hits. Third, we combined the two searches, resulting in 845 hits after duplications were omitted. We also examined the citations in the papers that were obtained, and conducted searches in Open grey literature (OpenGrey, PsycEXTRA and Home—ClinicalTrials.gov), resulting in 22 papers. Experts with clinical experiences and scientific publications on pain, fatigue or other relevant aspects of sHTADs were asked, but no additional papers were found. The search terms and search strategy is described in Table 1.

**Table 1:** Search strategy

The following search term were used:
*Search 1***:** Heritable thoracic aortic aneurysm and dissection OR heritable aortic disease OR hereditary thoracic aortic diseases OR familiar thoracic aortic aneurysm dissection OR genetic aortic diseases OR Marfan syndrome OR MFS OR Loeys-Dietz OR LDS OR vascular Ehlers Danlos syndrome OR vEDS OR EDS OR GAD OR Rienhoff OR arterial tortuosity OR dissection OR aneurysm OR osteoarthritis syndrome OR HTAAD OR HTAD OR FTAAD OR GAD OR the terms of the genes associated with HTADs (3) (resulting in 15.872 hits)
*Search 2*: Fatigue OR tiredness OR exhaustion OR dizziness OR sleep problems OR sleep apnea OR vitality (resulting in a total of 9547 hits)
*Search 1* and 2 were combined: Resulted in 845 hits (after removal of duplicates and publications in language not fulfilling the inclusion criteria (e.g., Chinese)
Search in grey literature and examining the references of included, resulting in 22 articles

*Eligibility criteria:* Articles were considered for inclusion if they investigated fatigue in sHTADs. The eligibility criteria were developed based on preliminary review of a random subset of relevant fatigue studies, a scoping review of fatigue in rare diseases [21], as well as theoretical literature about the concept of fatigue. The three basic inclusion criteria were as follows: (i) all individuals affected by a specific sHTAD as defined in the search criteria. Studies with mixed population including sHTADs that did not report subgroup analysis were excluded; (ii) all types of studies, peer-reviewed articles presenting own results, published in English, French, German, Norwegian, Danish or Swedish language; and (iii) where the aim and outcome of the studies “included examining self-perceived fatigue or vitality, and/or predicting variables or factors associated with fatigue/vitality in sHTADs” as a primary or secondary outcome.

No exclusions were made on the basis of age, gender or ethnicity. Unpublished data or case-report, conference abstract, posters, letter to editors, expert opinions, guideline, unpublished data and study protocols and studies with less than six participants were excluded.

#### Selection of publications

Two researchers (GV/HJ) independently reviewed the abstracts and/or articles from each publications that was identified through the search strategy described above. When considered potentially eligible, the full text of these studies was obtained and reviewed by the same two researchers against the eligibility criteria to determine their eligibility. A third (TB) and fourth researcher (AMG) verified the articles inclusion or exclusion in accordance with the final eligibility criteria.

#### Handling data, critical appraisal and data extraction

All included articles were screened and categorized independently by three researchers (GV, HJ, TB) on the basis of the content of the article [36, 39, 40]. Discrepancy and disagreement were discussed and resolved by involving a fourth researcher (AØG). The studies were first categorized according to which sHTADs the study dealt with. Specific validated criteria were used for critical appraisal of quantitative [39], qualitative [41] and review [42] articles. Seven criteria were used to evaluate the quantitative studies: (i) sample size (ii) sample representativeness (iii) control group (iv) the validity of the measurement (v) drop-out/missing data (vi) blindness and (vii) credibility assessment [39]. No controlled trials or randomized controlled trials (RCT) were found; therefore, the criterion about blindness was omitted. Six validated criteria were used for assessing the qualitative studies [41] and seven criteria for assessing reviews [42]. In addition to the validated criteria (questions) for critical appraisement of the different types of studies, we added two criteria (questions): To which degree the papers discussed the limitations of the study and the extent of contribution on new results about fatigue/vitality in sHTADs. Because of the complicated process leading to the diagnosis of sHTADs, we also collected information from the included articles about their use of diagnostic criteria and/or genetic testing to identify their study population. The quality assessment criteria for quantitative, qualitative and review studies and the justifications for the assessment of each article are reported in Additional file 3.

The studies were too heterogeneous to perform statistical pooling and meta-analyses. Therefore we performed a narrative synthesis of the findings, taking into account methodological quality and analytic rigor in the examination of the reported findings [42, 43].

A mixed method thematic analysis was conducted to structure and depict all variables involved in the reviewed studies [44]. All included articles were screened and categorized independently by two researchers (GV/HJ) on the basis of the of the content in the articles. Discrepancy and disagreement were discussed by involving a third (TB) or a fourth (AØG) researcher. Information was extracted on study population, diagnoses, recruitment sources, study designs, methodology, fatigue measurements and other validated instruments, key predictor variables, main results and authors’ conclusions. Each study was categorized according to whether fatigue/vitality was the primary (major) or secondary outcome. Using a matrix [44, 45] the key features were summarized and synthesized. The relationship between variables and between different levels and groups of variables (main-/sub-variables) were indicated. A mixed-methods approach [44] was used to integrate the conclusions from the qualitative and quantitative strands (comparing, contrasting building on or embedding with the other) in order of to provide a fuller understanding of fatigue in sHTADs. Finally, the results of the articles were synthesized and summarized in three different main themes (i) the prevalence of fatigue in sHTADs (ii) the associations/predictors of fatigue and (iii) fatigue in children and adolescents with sHTADs.

### Qualitative focus group interviews

The qualitative focus group study has been approved by the Regional Committee for Medical Research Ethics (Health Region South-East) (2017/745). The study was conducted according to the COREQ-checklist for qualitative research [46]. We have previously published an article with comprehensive description of methodology of the focus group interviews [12], therefore only a brief summary about the methodology is presented in this paper.

#### Study design and informants

Patients with a confirmed diagnosis MFS, LDS and vEDS, registered at TRS National Resource Center for Rare Diseases in Norway were eligible and were invited to participate between October 2017 and April 2018. A combination of convenience and purposive selection method was used. The intention was to include approximately 36 patients, as this was considered appropriate to capture unique variation and saturation of the data.

#### Strategies for ensuring trustworthiness and credibility

Three researchers (GV, HJ, TB) ensured trustworthiness and credibility throughout the data collection and analysis process [46, 47]. The study reporting adheres to the consolidating criteria for reporting qualitative research [46], and the Standards for Reporting Qualitative Research [47].

#### Procedures and analyses

A semi-structured interview-guide was developed as a framework for the focus group interviews. The interviews lasted for an average of 110 min (range 90–120 min) and were audiotaped and transcribed verbatim by two researchers (GV/HJ). An Inductive Systematic Text Analysis (ISTA) was conducted [48, 49]. To ensure the robustness of the study we followed the principles of Malterud [48, 49] of six step-by-step analysis (Additional file 4), based on the four criteria of credibility, dependability, confirmability and transferability [47, 49, 50]. Two researchers (GV/HJ) independently conducted the preliminary analysis and compared preliminary results. To assess the interpretative rigour of the analysis, we assessed inter-coder agreement to control the coding accuracy and monitor inter-coder reliability. Differences were discussed in an iterative process until consensus was reached among the research team (GV, HJ, TB, AØG).

## Results

### Systematic review results

#### Search results

**Fig. 1:**
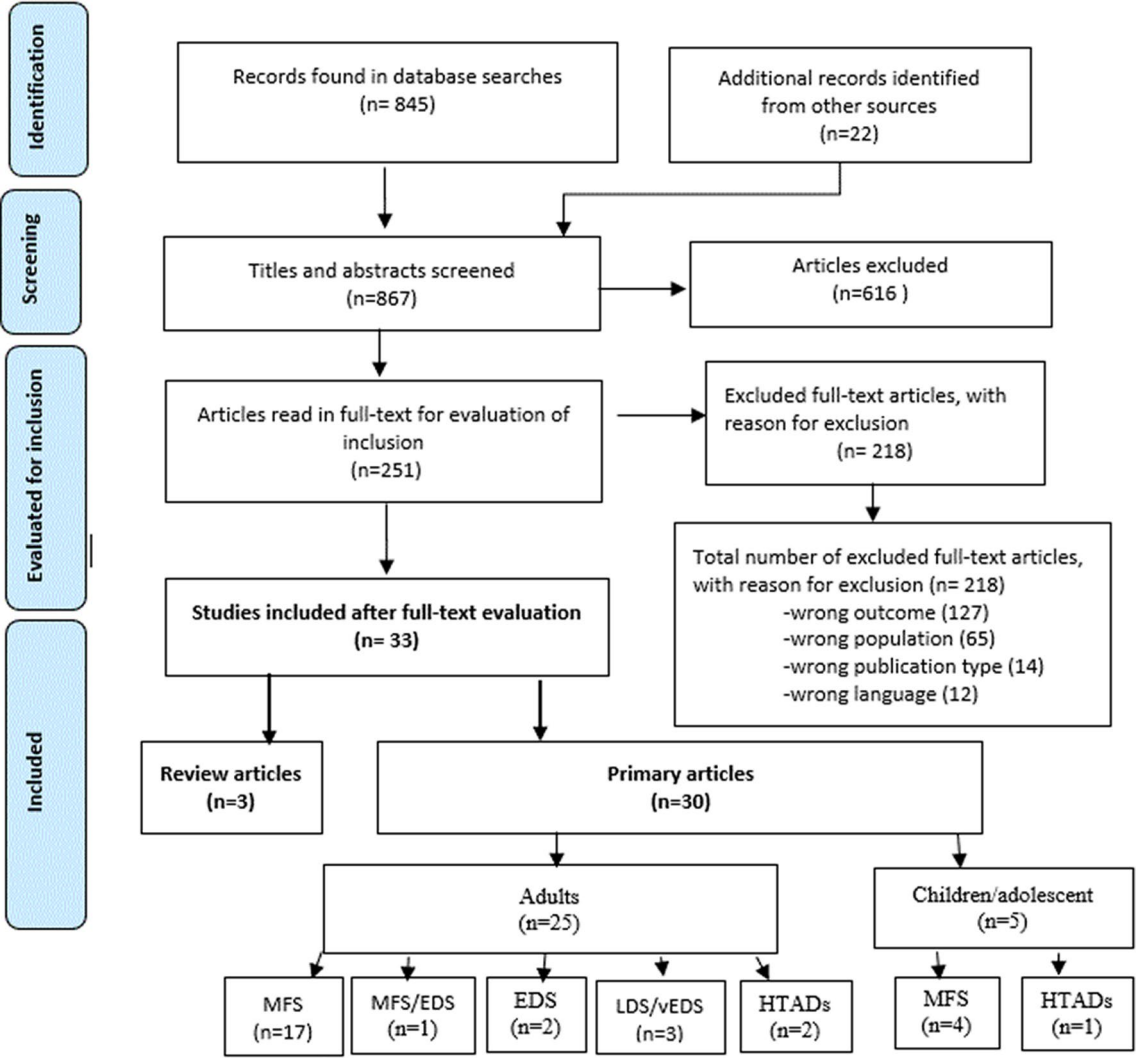
Flowchart of search, screening and inclusion process of the systematic review

The search strategy is presented in the flow chart in Fig. 1. A total of 867 articles were identified, 251 were read full text, of these 33 articles satisfied the eligibility criteria and were included in this review. There were 30 primary and three secondary (review) studies.

#### Included secondary studies: review articles

Three review articles fulfilled our inclusion criteria [19, 20, 51], but in no reviews the primary outcome was fatigue. Two review articles dealt with psychosocial aspects of MFS. One [20] included 11 articles dealing with fatigue, and the other [19] four articles. The last review article [51], addressing quality of life (QoL) in people with HTADs, included five articles of fatigue. All three review articles indicated that fatigue appears to be prevalent in patients with sHTADs, but none reported the estimated prevalence of fatigue of included studies. Nevertheless, all reported that fatigue in patients with MFS seems to have a huge impact on their QoL and daily life.

#### Included primary studies: fatigue in sHTADs

Of the 30 primary studies dealing with fatigue in different sHTADs, 25 dealt with adults while five with children/adolescents. Twenty-one articles on patients with MFS (17 on adults and 4 on children), one article on MFS and EDS, two on EDS, three on vEDS/LDS, and three on different sHTADs (two on adults/one on children). One third (n = 10) of the studies [52–61] did not describe if they included patients with verified diagnoses. The diagnoses were either self-reported or the confirmation of diagnoses was not reported in the study. The rest of the studies either included patients diagnosed according to the Ghent Nosology for MFS, or genetic verified diagnoses for LDS, vEDS and the other sHTADs. Eighty-three percent of the articles were published from 2010 to October 2022, with 70% published from 2015 onward. Twenty-three articles (77%) were from Europe, six (20%) from USA, and one (3%) from Korea (Table 2).

**Table 2:** Data extraction of included studies of patients with sHTADs

References: First authors, year, title, journal, country	The aim(s) of the study (as reported by the authors)	Methodology Study design, methods and outcome measures	Participants: Number (N), gender (G), age (A) diagnosis (D), recruitment location (R)	Results on fatigue/ vitality: Prevalence of severe fatigue/ reduced vitality, mean fatigue/ vitality score, associations to fatigue/ vitality, other aspects (patients experiences and perceptions)	Focus on fatigue: Primary/secondary outcome Conclusion Conclusion related to fatigue/vitality
*Marfan syndrome (adults)*					
[17] Bathen et al. 2014 Fatigue in adults with Marfan syndrome, occurrence and associations to pain and other factors American Journal of Medical Genetics, A Norway	To investigate fatigue in adults with MFS, fatigue levels and prevalence of severe fatigue, and associations to sociodemographic and medical aspects	Cross sectional quantitative questionnaire study: Study specific questions including validated instruments: Fatigue severity Scale (FSS) and Standardized Nordic questionnaire (pain)	N = 72 G = 57% females A = mean age: 44,2 y (20–71y) D = Marfan syndrome (Ghent 1) R = Clinic	42% severe fatigue and 29% borderline fatigue, 29% no fatigue.Significant higher fatigue than GP (FSS mean 4.72/4.0) (SD: 1.4/1.3). Cut-off ≥ 5, severe fatigue. No significant associations between fatigue score and MFS related health problems (i.e. aortic dilatation, aortic dissection, aortic surgery and visual problem due to lens dislocations), or use of blood pressure medications Fatigue significantly associated to pain and decreased work participation	Primary outcome The study confirms that fatigue affects persons with MFS by interfering with their daily life. Their level of fatigue and the prevalence of severe fatigue are higher than reported for both the general population and a study sample if RA patients, but lower than reported for groups of patients with other chronic condition. Multivariable regression analyses reveal that chronic pain and employment status show significant associations to fatigue. There is need for more research on fatigue in MFS
[71] Benninghoven et al. 2017 Inpatient rehabilitation for adult patients with Marfan syndrome: an observational pilot study Orphanet Journal of Rare diseases Germany	To confirm that our rehabilitation program was feasible and medically safe To apply standardized instruments to assess the impact of the rehabilitation program on physical fitness and psychological wellbeing	Pilot intervention study without controls. Measuring pre-and post-intervention (after 12 month). Study specific questionnaire including: Fatigue Severity Scale (FSS), Hospital Anxiety and Depression Scale (HADS) Somatization subscale of the Symptom Checklist-90-R, Short Form-36 (SF-36 vitality), Nottingham Health Profile, quality of life	N = 18 G = 71% females A = mean46,7 y (SD 7,8 y) D = Marfan syndrome (n = 17) (Q87.4 according to ICD10), Loeys Dietz (n = 1) R = German MFS self-help organisation and Clinic	Severe fatigue at admission was. (42%) Pre/post: FSS mean:5,62/4.92 (SD:0.91/1.51) Vitality mean: 34.7/42.3 (SD:13.9/21.0. Comparing within the study group Rehabilitation program had significantly positive impact on changes in fatigue	Secondary outcome This study found similar levels of fatigue in the pre-rehabilitation MFS population as Bathen et al. 2014 The three week rehabilitation program improved physical fitness and psychological wellbeing and fatigue one year after ended rehabilitation stay
[62] Fusar-Poli et al. 2008 Determinants of QoL in Marfan syndrome Psycosomatic Italia	To investigate QoL and demographic determinants influencing QoL in MFS,	Cross sectional quantitative questionnaire study: study specific questionnaire on sociodemographic, including validated instruments. Short Form -36 (SF-36 vitality), Karofsky index	N = 36 G = 56,7% female A = mean 31.73y D = MFS (Ghent 1) R = Clinic	SF-36 vitality mean score 61.67 (SD:17,23)). Refer for norm data = 100 of all SF-36 subscale Self-reported vitality was significantly lower than for the general population	Secondary outcome The article suggest a clinically significant relationship between MFS, psychosocial adjustment and mental quality of life
[52] Ghanta et al. 2016 Midterm suvival and quality of life after extent II Thoracoabdominal aortic repaid in Marfan syndrome The Annals of Thoracic Survey USA	To evaluate operative outcomes, midterm survival and QoL in patients with MFS who underwent extent II TAAA (Thoracoabdominal aortic aneurysm) repair	Cross sectional quantitative questionnaire study, Short Form-12 (SF-12, vitality sub score)	N = 49/of these 24 responded on SF-12 G = 35% female A = 76% were 50 y or less D = MFS underwent Crawford extent II TAAA repair. Criteria not described R = Clinic	SF-12 vitality mean score: 51.5 (SD not reported). Norm data for SF-12vt was 50	Secondary outcome Nothing in the conclusion about fatigue
[63] Moon et al. 2016 Structural equation modeling of the quality of life for patients with Marfan syndrome Health and Quality of life outcome, Korea	To build QOL structural model of patient with MFS, verify goodness of fit and determine the factors that affect the QOL	Cross sectional quantitative questionnaire study: study specific questionnaire including: Short Form 36 (SF-36 vitality sub score), Fatigue Severity Scale (FSS), the Hospital Anxiety and Depression scale (HADS), Body Image States Scale (BISS), pain analog scale (VAS),	N = 218 G = not reported A = ≥ 20 y D = MFS (Ghent 2) R = Clinic	FSS mean score: 46.3 (SD: ± 6.5), (results on GP/CG not reported) SF-36 vitality score not reported Greater fatigue was associated with lower QoL	Secondary outcome The study found that depression, anxiety fatigue, pain and body were bio-behavior variables that affect QoL Approaches should be developed for effectively managing bio-behavioral factors to improve QoL of patients with MFS
[72] Percheron et al. 2007 Muscle strength and body composition in adult women with Marfan syndrome Rheumatology France	To assess skeletal muscle function and body composition in a group of women with Marfan syndrome compared with matched controls After assessing daily physical activity levels and self-reported fatigue, lower-limb muscle strength and fatigue were measured under isokinetic and isometric conditions, and correlation between the results and lean leg mass determined using dual-energy X-ray absorptiometry (DEXA)	Prospective intervention study including controls Study specific questionnaire with validated instruments Fatigue Severity Scale (FSS), the International Physical Activity Questionnaire (IPAQ), Peak force testing and Isokinetic and Isometric strength testing	N = 21 (19 matched controls) G = 100% female A = 18–55y (20, 9–53,7y) D = MFS (free of major cardiovascular disease) (Ghent 1) R = Clinic	-FSS mean score: 5.11 (SD ± 1.18) Matched controls = 3.31. + 1.08:range 1.9–5.3. (P < 0.05) FSS scores indicated greater fatigue in the patients than in controls matched on age, gender and anthropometrics. Despite reports of greater fatigue in the MFS, there was no evidence of a difference in objective muscle fatigue between the two groups	Secondary outcome Fatigability is a major complaint of patients with MFS Authors claimed that they could not exclude that frequent complaints of subjective fatigue might reflect a limitation in aerobic capacity induced by beta-blocker treatment, likely through limitation in the cardiac output increase
[53] Peters et al. 2001. Living with Marfan syndrome I. Perceptions of the condition Clinical Genetics USA	To investigate clinical features and family history and their perceptions of the condition as conceptualized by the self-regulatory model of illness perception	Cross-sectional quantitative study, with study specific questionnaire Fatigue: Having fatigue or not? (yes/no) Illness perceptions; The Illness Perception Questionnaire, The Center for Epidemiological Studies Depression Scale	N = 174 G = 58% female A = mean age 38.9y D = MFS (not clinically verified diagnosis) R = Clinic (12,6%) and National Marfan Foundation (87.4%)	88.5% (n = 154) reported significant level of fatigue. Norm data not reported Significant positive association between being female, perception of MFS as lethal condition, and perception of MFS as serious condition	Secondary outcome Cardiovascular history, experiences with pain, fatigue, striae, and depression may play important roles in the formation of the subjective perception of MFS Fatigue has been associated with diminished cognitive functioning and the perception that MFS is a lethal condition
[31] Rand-Hendriksen et al. 2007 Fatigue, cognitive functioning and psychological distress in Marfan syndrome, a pilot study Psychology, Health and Medicine Norway	To assess self-reported fatigue, cognitive function and psychological distress, correlated to visual acuity, joint hypermobility and use of Beta-blockers	Cross sectional quantitative study with study specific questionnaire, including validated instruments: Fatigue Severity Scale (FSS), Fatigue Questionnaire (FQ), General Health Questionnaire 30 and a battery of neuropsychological testing	N = 16 G = 13 (81%) female A = 18–30y D = MFS (Ghent 1) R = Clinic	Mean FSS score: 4.7 (SD: 1.4). Norm data; GP = 2.3 (SD = 0.7) Mean FQ score 15.2 (SD: 4.98). Norm data from Loge et al. 1998 There was a significant inverse correlation between FQ rating and some neuropsychological tests. No correlation to use of Beta Blockers or hypermobility. Significantly higher amount of mental fatigue in MFS women Fatigue seems to be serious problems and seems related to some areas of cognitive functioning. Self-reported fatigue were comparable with fatigue reported in other severe chronic diseases, and was primarily in the mental/ psychological domains	Primary outcome The importance of considering mental fatigue as a major contributor to possible neurocognitive impairments is further stressed by significant correlation between mental fatigue and several of neuropsychological variables The interplay between fatigue, psychological distress and other psychological variables in MFS need further research
[13] Rand-Hendriksen et al. 2010 Health related quality of life in Marfan syndrome: A cross sectional study of Short Form 36 in 84 adults with a verified diagnosis Genetic in Medicine Norway	To explore quality of life as measured with SF-36 in adults with verified Marfan syndrome diagnoses, and potential association between SF-36 and presence of biomedical criteria and symptoms	Cross sectional quantitative study, with study specific questionnaire and medical examination, including Short form-36 (SF-36 vitality)	N = 84 G = 63% female A = mean 39,6y (19–69y) D = MFS (Ghent 1) R = Clinic	SF-36 vitality mean score: 40.0 (SD: 22.0). Matched control SF-36vt 61 No associations with gender or biomedical criteria or symptoms of MFS or any of the subscales in SF-36 The vitality score for people with MFS was significantly lower than for the general population	Secondary outcome Fatigue and reduced physical endurance, coping, stigma and pain, adherence to medication and restriction in physical activity may influence quality of life
[64] Rao et al. 2016 Quantifying Health Status and Function in Marfan Syndrome Journal of Surgical Orthopaedic Advances USA	To understand the self-perception of physical and mental well-being in patients with MFS compared to the general US population To quantitate quality of life and physical function and to focus on their levels and location of pain To document the effects of MFS on employment	Cross sectional quantitative questionnaire study with study specific questionnaire about demographics, MFS related health problems and fatigue (using VAS scale to rate importance—no validated instruments), and a validated instruments measuring quality of life; Short Form- 36 (SF-36 vitality)	N = 230 G = 58% female A = mean 44y (14 -82y) D = Marfan syndrome (Ghent 1, verified by genetics) R = Annual Meeting of Marfan Foundation	SF-36 vitality mean score female/male: 47.6/51.6 (SD: ± 24.7/20.8). Norm data US population:SF-36vt = 61 The patients’ sense of vitality to function are severely impaired compared to those of general population because of pain, cardiac and back involvement, fatigue and poor physical function. Fatigue, likely also affects patients ability to cope with daily activities, including integration into work and social life	Secondary outcome The cause of fatigue is likely multifactorial: the multisystem organ involvement and high prevalence of specific and general pain directly contributed to lower energy level and sense of well-being
[54] Ratiu et al. 2018 Executive function and quality of life in individuals with Marfan syndrome Quality of Life Research USA	This study examined perceptions of executive function and QoL among persons with MFS	Cross sectional quantitative questionnaire study with study specific questionnaire of sociodemographic questions (Webexec) and the Quality of Life Index	N = 318 G = 208 females, 104 males, 1 transgender, 5 not reported A = mean 41,4y (18–86y) D = MFS (not verified diagnosis) R = Symposium National Marfan Associations	Mental fatigue was the strongest predictor of total QoL No comparison data reported The study demonstrated that patients with MFS may experience specific difficulties in executive function, such as mental fatigue leading to diminished QoL	Secondary outcome Mental fatigue and commitment may account for total QoL and satisfaction with life
[65] Schoormans et al. 2012 Mental quality of life related to cytokine genetic pathway PLOS one The Netherland	To explore whether genetic variability and activity contributes to QoL in patients with Marfan syndrome, a genetic connective tissue disorder	Cross sectional quantitative questionnaire study: study specific questionnaire including Short Form-36 (SF-36 vitality)	N = 121 G = 33% female A = mean 37y D = MFS (Ghent 1) R = Clinic	SF-36 vitality mean score not reported Post-hoc analysis of systemic inflammatory mediators showed that patients with lowest Mental component score and vitality scores had high levels of CCL11 cytokine. Both mental QoL and vitality were independently, negatively related to CXCL9 and CXCL11 expression levels Additionally, in our patient population vitality was negatively related to the IFNA6 gene Overall, mental QoL was normal. 10% of patients had low scores for Mental component and vitality No comparison data reported	Secondary outcome Knowledge about this genetic component of QoL provides insight and can eventually allow us to identify patients susceptible to poor QoL Patients with low vitality scores had high expression levels of CXCL9, CXCL11 and IFNA6 cytokine-related genes independent of patient characteristics
[32] Van Andel et al. 2022 A cross sectional study on fatigue, anxiety and symptoms of depression and their relation with medical status in adults patients with Marfan syndrome Clinical Genetics Netherland	To determine prevalence of fatigue, anxiety, and symptoms of depression in MFS patients, and to assess the degree to which sociodemographic and clinical variables are associated with fatigue and psychological aspects	Cross sectional quantitative questionnaire study on sociodemographic aspect and clinical examination, including self-reported validated instruments: The Checklist Individual Strength (CIS), Hospital Anxiety and Depression scale (HADS)	N = 155 G = 49% female A = 31,51y, mean 42y D = Marfan syndrome (Ghent2) R = Clinic	37% experienced fatigue, significantly higher than GP MFS/GP: CIS mean (SD):31,9 ± 8 23.0 ± 10.8 (Cut-off score > 35 for severe fatigue,) Severe fatigue associated with being female, aortic surgery and chronic pain. Using Losartan was associated with less severe fatigue and beta-blockers not associated with severe fatigue	Primary outcome MFS patients reported significantly higher fatigue than GP. Since the cause of fatigue is unclear, more research is needed Indication that losartan was negatively associated with fatigue, psychological distress can be a cause of fatigue, mitochondrial dysfunction can result in lack of energy, and pain may be difficult to distinguish from fatigue
[55] Van Dijk et al. 2008 Is fatigue in Marfan syndrome related to orthostatic intolerance? Clinical Autonomic Research The Netherland	To investigate the relationship between symptoms of orthostatic tolerance and fatigue in patient with MFS, whether termination of beta-blockers therapy improves orthostatic tolerance	Prospective study Study 1: A study specific questionnaire were used to assess symptoms of orthostatic tolerance, including validated instrument of fatigue MFI-20 Study 2: orthostatic tolerance with and without beta-blockers was assessed in a physical experiment	N = 58 (study 1:n = 49/study 2, n = 9) G = 34% /44% female A = mean 35y (23–68y)/36y (20–50y) D = MFS (Ghent 1 for those using beta-blockers, the rest not described) R = Clinic	MFS population compared to general population (MFI-20): General fatigue mean score:13.3/9.9 (< 0.01) Physical fatigue, mean score: 12.6/8.8 (< 0.1) Reduced activity mean score:11.2/8.7 (< 0.01) Reduced motivation, mean score:9.9/8.2 (8.9/4.0) 0.018 Mental fatigue mean score:9.6/8.3 (0.10) Significant correlation between orthostatic tolerance and fatigue (all MFI-20 scales) were found	Primary outcome Patients with MFS have more complaints of fatigue and orthostatic intolerance than the general population There was no significant relationship between fatigue and B-blockers use, but note a significant relationship between fatigue and orthostatic intolerance
[73] Vanem et al. 2021 Health related quality in life in Marfan syndrome: a 10 years follow-up Health and Quality of Life outcome Norway	The aim of this 10-year follow-up study was to assess changes in the eight subscales of the SF-36 and changes in mental and physical component score. Secondly, to explore whether age, severe organ pathology predict decline in any of the subscales	Prospective longitudinal quantitative clinical questionnaire study including: Short Form-36 (SF-36 vitality)	N = 47 G = 72.3% (n = 34) female A = mean 49,9y D = MFS Ghent 1 R = Clinic	SF-36 vitality mean score baseline/norm score: 44.0/43.9 (SD:18.8/8.9) Follow up/norm score = 42.7/43.3 (SD:19.2/9.1) Significantly reduced scores in all sub scales except mental health (vitality z-score -0.76) than GP	Secondary outcome Lower scores in all the domains of the SF-36 compared to the reference population, with a stabile vitality score over 10 years
[66] Velvin et al. 2015 Work participation in adults with Marfan syndrome: Demographic characteristics, MFS related health symptoms, chronic pain, and fatigue American Journal of Medical Genetic, A Norway	To investigate work participation and explore the associations between health related consequences of MFS and other factors, on work participation. In addition: describe the prevalence of health problems in young adults compared to older adults with MFS	Cross sectional quantitative study with study specific questionnaire, including validated instruments Fatigue Severity Scale (FSS); Nordic questionnaire, battery of questions from National Labor force survey	N = 70 G = 57% female A = mean 43y (20–67y) D = MFS (Ghent 1) R = Clinic	42% had severe fatigue (cut-off score ≥ 5) Higher levels of fatigue were associated with earlier retiring from work	Secondary outcome Fatigue is found to play a major role in daily life for many people with hereditary connective tissue disorders such as MFS
[67] Velvin et al. 2016 Satisfaction with life in adults with Marfan syndrome: associations with health-related consequences of MFS, pain, fatigue, and demographic factors Quality of Life Research Norway	To examining satisfaction with life (SWL) in adults with MFS compared to the general Norwegian population and other patient groups. Exploring the associations between SWL and demographic characteristics, contact with health and social services, MFS-related health problems, chronic pain, and fatigue	Cross sectional quantitative study with study specific questionnaire, including validated instruments measuring. Fatigue Severity Scale (FSS); Nordic questionnaire, Satisfaction with Life Scale (SWLS)	N = 72 G = 57% female A = mean 44,2y (20–72y) D = MFS(Ghent 1) R = Clinic	42% had severe fatigue. (cut-off score ≥ 5) Fatigue were significantly associated with lower satisfaction with life scores	Secondary outcome Patients with MFS who experience higher levels of fatigue report lower satisfaction with life Severe fatigue and aortic dissection are the problems that had greatest negative impact on life satisfaction
*Ehlers Danlos syndrome (adults)*					
[68] Schubart et al. 2019 Cluster Analysis to Delineate Symptom Profiles in an Ehlers Danlos Syndrome Patient Population Journal of Pain and Symptom Management USA	To seek evidence of phenotypic subgroups of patients with distinctive symptom profiles and describe these resulting subgroups	Cross sectional quantitative design. Data were extracted from the National Institute on Aging Intramural Research Program. Study specific questionnaire and validated instruments: the Wisconsin Brief Pain Inventory. Physical Fatigue (PF), the Multidimensional Fatigue Inventory (MFI), the Epworth Sleepiness Scale, Short Form-36 (SF-36 vitality)	N = 175 G = 77% female A = mean 42 y, (21y ≥) D = EDS; Classical (26 patients), Hypermobile (34 patients), Vascular (51 patients), or Rare and Unclassified (64 patients). (clinical examination, but diagnostic criteria not described) R = Clinic, from protocol to study	Mental Fatigue was more likely to occur in Vascular EDS (47%) and Rare/Unclassified (42%) compared to Classical (23%) and Hypermobile (24%) The Mental Fatigue subgroup was characterized by a higher mean value for mental fatigue and daytime sleepiness relative to the other variables High Symptom Burden group contained 41%) (n = 71) and Mental Fatigue group contained 37% (n = 65)	Primary outcome The heterogeneous nature of EDS, with overlapping symptoms between subtypes and a wide divergence in degree of symptoms within subtypes was conformed The Mental Fatigue subgroup (65 patients) had a high mean value for mental fatigue and daytime sleepiness, but a lower mean value for pain Mental Fatigue was more likely to occur in Vascular type and Rare/Unclassified type compared to Classical and Hypermobile type
[57] Voermans et al. 2010. Fatigue is a frequent and clinically relevant problem in EhlersDanlos Syndrome Journal of Pain and Symptom management The Netherlands	To investigated prevalence and impact of fatigue and associated features in a large group of EDS patients	Cross sectional quantitative study with study specific questionnaire including validated instruments measuring fatigue; Checklist Individual Strength subscale fatigue (CIS), pain; the McGill Pain Questionnaire, functional impairment; the Sickness Impact Profile, sleep disturbance; the Symptom Checklist subscale, Short Form 36 (SF-36 vitality)	N = 273 G = 91% female A = mean 41y (16–89y) D = Self-reported Ehlers Danlos syndrome sub-groups; EDS classic type (n = 45), hypermobility type (n = 162), vascular type (n = 11), kyphoscoliotic type (n = 2,) unknown (n = 53), (not medically verified diagnosis) R = Members of the Dutch patient organization of EDS 19 patients from clinic	Classified in a severe fatigue group (vs. no fatigued group): EDS (69%), hEDS (84%), vEDS (5%) Cut-off score of CIS was 35 for severe fatigue Multiple regression analysis of data of all patients resulted in a model in which pain severity (most severe pain [VAS]) and fatigue severity predicted 31% of functional impairment Severe pain was significantly associated with severe fatigue. Pain contributes to functional impairment in daily life, independent of the level of fatigue	Primary outcome 77% of EDS patients suffered from severe fatigue, and patients who were severely fatigued were more impaired and reported higher level of psychological distress Patients with hEDS were most often severely fatigued Severe fatigue in EDS was related to sleep disturbances, concentration problems, social functioning, self-efficacy concerning fatigue and pain
*Marfan syndrome (MFS) and Ehlers Danlos syndrome (EDS) (adults)*					
[56] Verbraecken et al. 2001 Evaluation for sleep apnea in patients with Marfan syndrome and Ehlers Danlos syndrome Clinical Genetics Netherlands	To examine the exact nature of sleep complaint in these patient groups	Cross sectional quantitative questionnaire study, with study specific questionnaire including the Short Form-36( SF-36 vitality)	N = 24 (MFS = 15, EDS = 9) G = 62% (n = 15) female A = mean 33/34y D = MFS, not verified diagnosis of MFS/EDS R = Annual meeting of Marfan Association	SF-36 vitality mean score: MFS = 62 (SD ± 7), EDS = 54 (SD ± 5) Healthy controls = 75, SD ± 2 Physical function correlated with the presence of fatigue in the morning and with hypersomnolence Vitality significant lower than for general population 75 (± 2)	Secondary outcome Data revealed that individual with MFS and EDS experience frequent sleep complaints, which are likely due to pain and sleep apnea in MFS, while EDS patients more frequently suffer from severe pain and periodic limb movements
*Vascular Ehlers Danlos (vEDS) Syndrome and Loeys Dietz syndrome (LDS) (adults)*					
[11] Johansen et al. 2020 Adults with Loeys–Dietz syndrome and vascular Ehlers–Danlos syndrome: A cross-sectional study of patients experiences with physical activity Journal of Disability & Rehabilitation Norway	To study patients perception of physical activity in patients with Loeys–Dietz syndrome and vascular Ehlers–Danlos syndrome	Cross sectional quantitative study, with study specific questionnaire including validated instruments Fatigue Severity Scale (FSS), The Hospital Anxiety & Depression Scale (HADS), pain: one item from Standardized Nordic Questionnaire	N = 52 G = 58% female A = 18–68y, mean 43.5y D = 18 vEDS, 34 LDS (genetic verified diagnosis) R = Clinic	58% severe fatigue, mean FSS score was 4,8 (SD:1.6) Cut-off score ≥ 5 Physical Activity level were significantly negatively associated with fatigue (FSS) and anxiety (HADS-A)	Secondary outcome The findings indicate that persons with lower fatigue and lower anxiety scores reported higher physical activity level
[69] Johansen et al. 2021 Adults with Loeys-Dietz syndrome and vascular Ehlers-Danlos syndrome: A cross-sectional study of life satisfaction Journal of rehabilitation Medicine Norway	To explore self-reports of overall and domain-specific life satisfaction in adults with verified LDS or vEDS. To investigate the extent to which demographic and clinical factors are associated with different domains for life satisfaction	Cross sectional quantitative study, with study specific questionnaire including validated instruments: Fatigue Severity Scale (FSS), pain; one item from Standardized Nordic Questionnaire, psychological aspects; The Hospital Anxiety & Depression Scale (HADS) and Life Satisfaction 11 items	N = 52 G = 58% female A = 18–68 y, mean 43.5y D = 18 vEDS, 34 LDS (genetic verified diagnosis) R = Clinic	58% had severe fatigue Cut-off score ≥ 5 Low overall satisfaction, low satisfaction with health, leisure and vocation domains was significantly associated with severe fatigue	Secondary outcome Severe fatigue was found to be significantly associated with dissatisfaction in several life domains
[16] Johansen et al. 2022 Pain and fatigue in adults with Loeys-Dietz syndrome and vascular Ehlers-Danlos syndrome, a questionnaire based study American Journal of Medical Genetics A Norway	To present a more detailed description of self-reported chronic pain (intensity, locations, and perceived impact on daily life) and fatigue. Another aim was to explore the associations between chronic pain and fatigue with demographic- and clinical factors in adults with LDS and vEDS	Cross sectional quantitative study, with study specific questionnaire including validated instruments: Fatigue Severity Scale (FSS), pain; one item from Standardized Nordic Questionnaire, psychological aspects; The Hospital Anxiety & Depression Scale (HADS)	N = 52 G = 58% female A = 18–68y, mean 43.5y D = 18 vEDS, 34 LDS (genetic verified diagnosis) R = Clinic	58% severe fatigue. (LDS = 64%, and vEDS = 42%) (Norm data = 23%) Cut-off score ≥ 5) Mean FSS total score was 4,8 (SD:1.6) LDS/vEDS 5.1/4.3 (SD:1.5/1.6) Physical activity level, sleeping problems, chronic pain, cardiovascular and multi-organ burden and HADS-A were found to be statistically significantly associated with fatigue Disease burden, sleep problems, anxiety, chronic pain and fatigue seemed to mutually reinforce each other	Primary outcome Chronic pain and fatigue should be recognized as important features among patients with HTADs in the lifelong follow-up Clinical and research initiatives should consider interventions aimed at postponing the onset and/or reducing symptoms of pain, fatigue and sleep problems and thus reduce the total disease burden
*sHTAD (MFS, LDS, EDS, other sHTAD) (adults)*					
[58] Thijssen et al. 2020 Male and female differences in quality of life and coping style of patients with Marfan syndrome and hereditary thoracic aortic diseases Journal of Genetic Counselors Netherlands	To evaluate HRQoL in HTAD patients compared to the general population: assess female-male differences in HRQoL and factors associated with HRQoL evaluate coping styles in male and female HTAD patients	Cross-sectional quantitative questionnaire study: study specific questionnaire on sociodemographic including: Short Form 36 (SF-36 vitality), the Hospital Anxiety and Depression Scale (HADS), The Nijmegen Clinical Screening Instrument (NCSI)	N = 76 completed SF-36 (vitality) (total n = 142) G = 47,3% females A = mean 42.2y D = MFS (86,5%), LDS (5,6%), ACTA 2 (0,7%), others (3,5%), none (3,5%) R = Clinic	Mean SF-36 vitality of male 58.3 (± 204), Norm data = 71.9(± 18.2) SF-36 vitality of female 57.8 (± 21.3). Norm data = 64.3 ± 19.7) HTAD male had significant lower vitality than the general male population 71.9 (± 18.3).There was no significant differences between HTAD female and female in the general population 64.3 (± 19.7)	Secondary outcome Fatigue seems to be associated with less favorable scores on General Quality of Life
[12] Velvin et al. 2019 Physical Exercise for people with hereditary thoracic aortic diseases. A study of the patients perspectives Journal of Disability and Rehabilitation Norway	To explore the patients perspective on physical activity and exercise in patients with HTAD	Qualitative cross-sectional study: focus group interview	N = 36 G = 56% (n = 20) female A = Mean 48y (20–71y) D = Marfan syndrome (n = 14), Loeys-Dietz syndrome (n = 11), vascular Ehlers Danlos Syndrome (n = 11) R = Clinic	The participants described how fatigue and physical activity mutually negatively influenced each other Patients described how they tried to “recharge their battery” by mapping daily workload and planning daily activities that could conserve energy and reduce debilitating fatigue	Secondary outcome Timely information and physical education about possibilities and enjoyable activities is recommended and these patients may benefit from specialized rehabilitation, for decreasing fatigue
*Children/adolescents (n* = *5)*					
[59] Kelleher et al. 2015 Marfan syndrome patients experiences as ascertained through postings on social media sited American Journal of Medical Genetict USA	To investigate social media content related to Marfan syndrome	Qualitative descriptive document analyses for investigate public social media content, across six social media sites: Instagram, Pinterest, Reddit, Tumblr, Twitter and YouTube A codebook was developed using an iterative process to categorize posts and comments	N = 147 original posts G = Not described¨ A = Not described D = MFS (not verified) R = Marfan Foundation site for communication	Prevalence of posted comments of fatigue was 2% (3 of 147) on the internet Quotes like “I get tired easily”, “I get fatigue after small things like walking up stairs, or walking to the school” Tumblr was the only website where tired/fatigue was mentioned	Secondary outcome Fatigue/tiredness was observed as postings, and could be related to symptoms such as depression, cardiac or musculoskeletal involvement and represent areas that health care providers need to address when caring for patients with MFS
[60] Warnink-Kavelaars et al. 2019a Marfan syndrome in childhood: parents' perspectives of the impact on daily functioning of children, parents and family; a qualitative study BMC Pediatric Netherlands	To explore parents’ perspectives on the impact of MFS on daily functioning of children with MFS aged 4–12 years, themselves and family regarding functional performance, activities, participation, personal and environmental factors, and disease burden	Cross-sectional qualitative study with semi structured interviews and 3 focus group interviews, and interpretation by thematic analyses	N = 26 (10 individual interviews/16 focus groups) G = 60%/56% female A = 4–12y (and parents) D = Marfan syndrome (verification of the diagnosis- not described) R = Clinic	Parents reported that their children could not keep up with peers because of fatigue, pain and physical impairments Children experienced participation restrictions in school, sports, play and other leisure activities	Secondary outcome Professionals should address families their support needs and provide tailored interventions, rehabilitation and/or educational programs to empower and improve daily functioning of the children, parents and family
[74] Warnink-Kavelaars et al. 2019 Marfan in adolescent -adolescent perspective on physical function, disability and contextual factors and support needs European Journal of Pediatrics The Netherlands	To explore adolescents perceived impact of MFS on physical function, disability, contextual factors and supported needs	Cross sectional qualitative study, individual interview with semi-structured interview guide, with thematic analyses	N = 19 G = 12 male (7 female) A = 12–17y, mean 14,5y D = MFS (FBN1 confirmed) R = Clinic	Fatigue and pain limited school pace. Limitations in sport and actives due to fatigue and pain and fear of muscular and skeletal problems Adolescents described themselves as different due to fatigue, pain and appearance	Secondary outcome Difficulties in keeping up with their peers in social activities They asked for advices and support about improvement of fatigue, pain and physical impairment
[70] Warnink-Kavelaars et al. 2021 Parenting a child with Marfan syndrome: Distress and everyday problems Journal of Medical Genetics A The Netherlands	To assess distress and everyday problems of mothers and fathers with and without MFS, of a child with MFS, and child reported pain and fatigue	Cross-sectional quantitative questionnaire study online: Study specific questionnaire on sociodemographic aspect, included validated instrument: Distress Thermometer for Parents (T-P), Patients Reported Outcome Measure (PROM-portal)	N = 43 children, 42 mothers(29% with MFS), 26 fathers (60% with MFS) G = 44% of the children were female A = mean 8.9y (0.4 to 17.1y) D = MFS diagnosed according to the revised Ghent criteria R = Clinic	44% of the children were reported with sometimes/often fatigue, and with pain in 23.3% MFS mother vs healthy controls: 35.7% vs 55.7% MFS fathers vs healthy controls: 40.1% vs 44.1% There were no significant associations of parents distress and the child`s reported fatigue and pain	Secondary outcome Parents to a child with MFS did not show more clinical distress compare to parents of healthy children, (reported in 1/3 of the parents) but may increase in case with acute medical complications Advising monitoring distress in patens to child with MFS
[61] Warnink-Kavelaars et al. 2021 Heritable Connective Tissue Disorders in Childhood: Increased Fatigue, Pain, Disability and Decreased General Health Genes The Netherland and Belgium	To gain better insight into the prevalence and severity of fatigue, pain, disability and general health in children and adolescents diagnosed with the most common HCTD using standardized validated questionnaire	Cross sectional quantitative observational multicenter questionnaire study—A study specific questionnaire of sociodemographic aspects, including Patients Reporting Outcome Measures Information System (PROMIS) Fatigue 10a Pediatric v20 short form and Fatigue 10a Parent Proxy v2.0 short form, Child Health Assessment Questionnaire CHAQ, Pain Vas and General Health VAS	N = 107 G = 45% female A = mean 10.0y (14–18 y) D = MFS (n = 62/58%), LDS (n = 7/7%), EDS (n = 9/8%) hEDS (n = 29/27%) –criteria not described R = Clinic	Compared to normative T-scores—the HCTD-group no increased fatigue. EDS significantly increased fatigue, MFS decreased fatigue Norm data T-core mean (SD)50(10) MFS = 48 (11) LDS = 50(9) EDS = 56(13) hEDS = 63(8) Fatigue was significantly positively associated with disability, general health and pain	Primary outcome Compared to norm data, only children with hEDS reported increased fatigue, not children with MFS The new knowledge calls for monitoring and standardized assessment of fatigue, pain, disability and general health for these patient groups
*Review articles (n* = *3)*					
[20] Nielsen et al. 2019 A Review of psychosocial factors of Marfan syndrome: Adolescents, Adults, Families and Providers Journal of Pediatric Genetic USA	To review the current literature on psychosocial implication of MFS and other contributing factors that affect children and adolescents, adults and their families	Literature review, no quality assessment of the included articles	N = 11 (of 41) articles included outcome on fatigue	The results indicated that fatigue in MFS can impact work participation, QoL, and can be positively associated with pain and medication	Secondary outcome The findings regarding fatigue in the MFS population is somewhat inconsistent. Particular discrepancy of results on fatigue is associated with medication
[19] Velvin et al. 2015 Systematic review of the psychosocial aspects of living with Marfan syndrome Clinical Genetics Norway	To explore, critically appraise and to synthesize the literature of psychosocial aspects of Marfan syndrome	Systematic review, including validated criteria for appraisal the literature	N = 4 (of 15) articles included outcome on fatigue	Results indicate that higher fatigue was associated with female, psychological distress work disability and decreased quality of life	Secondary outcome Studies indicate that self-reported fatigue should be regarded as part of a distress/ fatigue complex rather than the physical consequences
[51] Velvin et al. 2019 Systematic review of quality of life in persons with hereditary thoracic aortic aneurysm and dissection diagnoses Clinical Genetics Norway	To explore, critically appraise and synthesize the literature on quality of life in patients with different HTAD diagnosis	Systematic review, including validated criteria for appraisal the literature	N = 5 (of 20) articles included outcome on fatigue	Studies found that fatigue was associated with decreased quality of life	Secondary outcome Pain, fatigue, psychosocial distress and learning disability may have greater impact of QoL than the objective biomedical findings

#### Methodological appraisal of primary studies

*Study design and level of focus on fatigue:* Of the 30 primary articles 22 studies were cross-sectional quantitative questionnaire studies [11, 13, 16, 17, 31, 32, 52–54, 56–58, 61–70]. Four were prospective: one pilot rehabilitation intervention study [71], two experimental studies [55, 72] and one longitudinal study [73]. Four studies were qualitative and used different methods: individual interviews [74], focus groups [12], document analyses [59] and combining individual interviews with focus groups [60]. Three of the qualitative studies dealt with children/adolescents [59, 60, 74]. No randomized controlled studies were identified.

In only eight [16, 17, 31, 32, 55, 57, 61, 68] of the 30 articles the primary outcome was to investigate fatigue. All these were cross-sectional quantitative studies. Four were on MFS, two on EDS, one on vEDS/LDS, and one on children with different sHTADs (MFS, vEDS) (Table 3).

**Table 3:** Quality assessment om included studies; quantitative studies, qualitative studies and review studies

Quantitative studies	
**Quality assessment criteria:** 1. Is the study design identified and appropriate? 2. How representative are the study group for the population? 3. Is there adequate control group? 4. Is the validity for measurement acceptable? 5. Is the study complete with regard to dropout/missing data and reporting respond rate? 6. Do the authors describe and discuss limitations with the study? 7. To what extent are study results influenced by factors that negatively impact their credibility? 8. Does the study contribute to (new) knowledge about fatigue in HTAAD?	Ratings: Very good, Good, Acceptable, Fair and Poor

Authors Years	HTAAD diagnosis	1. Study design	2. Representative sample	3. Control groups	4. Fatigue measure validity	5. Dropout/ missing data	6. Discuss limitations	7. Credibility	8. Novel knowledge about fatigue
*Adults*									
*Marfan syndrome (MFS)*									
[17] Bathen et al. 2014	MFS: verified Ghent 1	Good	Good	Good	Good	Good	Good	Good	Very good/good
[71] Benninghoven et al. 2017	MFS and one LDS: Verified diagnoses	Good	Acceptable	Good	Good	Good	Very good	Good	Very good/good
[62] Fusar Poli et al. 2008	MFS: All verified diagnosis	Good	Good	Acceptable	Good	Acceptable/good	Acceptable	Good	Acceptable
[52] Ghanta et al. 2015	MFS: 22 of 49 had verified diagnosis	Good	Acceptable	Good	Acceptable	Good	Good	Acceptable	Acceptable/good
[63] Moon et al. 2016	MFS: All verified diagnosis	Very good	Good	Good	Good/very good	Good	Very good/good	Very good	Good/very good
[72] Percheron et al. 2007	MFS All verified diagnosis,	Good/acceptable	Acceptable	Good	Acceptable	Good	Good	Good	Good
[53] Peters et al. 2001	MFS: only self-reported diagnosis	Good	Acceptable	Good	Acceptable	Good	Good/very good	Good,	Good
[31] Rand-Hendriksen et al. 2007	MFS, All verified diagnosis	Good	Acceptable	Good	Good	Good	Acceptable	Good	Very good
[13] Rand Hendriksen 2010	MFS: All had verified MFS,	Good	Good	Very good	Good	Good	Acceptable	Good	Acceptable
[64] Rao et al. 2016	MFS: All had verified diagnosis	Very good	Very good	Good	Acceptable	Acceptable	Fair	Acceptable	Good
[54] Ratiue et al. 2018	MFS: Self-reported diagnosis	Good	Acceptable	Acceptable	Acceptable	Acceptable	Very good-	Acceptable/good	Good/acceptable
[65] Schoorman et al. 2012	MFS All had verified diagnosis	Very good	Good	Good	Good	Good	Very good	Very good	Good
[32] Van Andel et al. 2022	MFS: All had verified diagnosis	Very good	Good	Good	Very good	Good	Good/very good	Good	Very good
[55] Van Dijk et al. 2008	MFS: Verified for some, but not all	Good	Acceptable	Good	Good	Acceptable	Very good	Good	Good
[73] Vanem et al. 2021	MFS: All had verified diagnosis	Very Good	Good	Very good	Good	Very good	Very good/good	Very good	Acceptable
[66] Velvin et al. 2015	MFS All had verified diagnosis	Good	Good	Good	Good	Acceptable	Fair	Acceptable-	Acceptable
[67] Velvin et al. 2016	MFS All had verified diagnosis	Good	Good	Good	Good	Good	Good	Good	Good
*Ehlers Danlos syndrome*									
[68] Schubart et al. 2019	HCTP Verified diagnosis, criteria 2015	Acceptable	Good	Good	Good	Good	Good	Good	Acceptable
[57] Voermans et al. 2010	EDS: Several subgroups of EDS, without medically verified diagnoses	Good	Acceptable	Good	Good/acceptable	Good	Good	Good	Good
*Marfan syndrome and Ehlers Danlos syndrome*									
[56] Verbraecken et al. 2001	MFS/EDS: Self-reported diagnosis	Good	Acceptable	Good	Good	Acceptable,	Good	Good	Acceptable
*Vascular Ehlers Danlos (vEDS) Syndrome and Loeys Dietz syndrome (LDS)*									
[11] Johansen et al. 2019	LDS/vEDS: All verified diagnosis,	Good	Good	Acceptable	Good	Good	Good	Good	Good
[69] Johansen et al. 2021	LDS/vEDS: All verified diagnosis,	Good	Good	Good	Good	Good	Good	Good	Good
[16] Johansen et al. 2022	LDS/vEDS: All verified diagnosis	Good	Good	Good	Good	Good	Good	Good	Very Good
*Different HTAAD diseases (including LDS, MFS and other HTAADs)*									
[58] Thijssen et al. 2020	HTAADs: 86,5% verified diagnoses	Good	Good-	Good	Good	Good	Good	Good	Good
*Children/Adolescents*									
[70] Warnink-Kavelaars, et al. 2020	MFS: All children had verified diagnosis,	Good	Good	Good	Good	Acceptable/good	Good	Good	Good
[61] Warnink-Kavelaars, et al. 2021	MFS, LDS, EDS and hEDS: Unclear if the children had verified diagnosis	Good	Acceptable	Good	Good	Good	Good	Good-	Very good

Qualitative studies	
Quality assessment criteria: 1. Is the research questions(s) clearly and explicitly stated? 2. How was the participants selected (described selection process)? 3. The researchers role and has it been taken in account? 4. Is the method appropriate for collecting data? 5. Is the method appropriate for analyzing the data and for ensuring scientific rigor? 6. The credibility of the study ( as a whole) 7. Do the study contribute to novel knowledge on the particular issue (fatigue)?	Ratings: Very good, Good, Acceptable, Fair and Poor

Author, year	Diagnosis (es)	1.Research question (s)	2.Recruitment	3.The role of researcher (s)	4.Appropriate method	5.Appropriate analysis (es)	6.Limitations	7.Credibility	8.Contribute to new knowledge 8
Adults									
[12] Velvin et al. 2021	MFS, LDS;, vEDS All verified diagnoses	Good	Good	Good	Good	Good	Good	Good	Acceptable
*Children/adolescents*									
[59] Kelleher et al. 2015	MFS: Not verified diagnoses, only self-reported	Good	Acceptable	Acceptable	Very Good	Good	Good	Good	Good
[74] Warnink-Kavelaars, et al. 2019	MFS: All had confirmed diagnosis	Very good	Good	Acceptable	Very good	Very good	Very good	Very good	Very good
[60] Warnink-Kavelaars, et al. 2019	MFS: No description of verified diagnosis	Good	Good	Acceptable	Very good	Good	Very good	Good	Good

Review articles	
**Quality assessment criteria:** 1. Is the review question clearly and explicitly stated? 2. Were the inclusion criteria appropriate for the review question? 3. Was the search strategy appropriate? 4. Were the criteria for appraising studies appropriate? 5. Was critical appraisal conducted by two or more reviewers independently? 6. Were there methods to minimize errors in data extraction? 7. Were the methods used to combine studies appropriate? 8. How is the credibility of the study? (limitations described, transparency, method, analyses and total impression) 9. Contribution to new knowledge on fatigue in HTAADs? (benefits worth the harms and costs), implication for practice and recommendation for further research	Ratings: Very good, Good, Acceptable, Fair and Poor

Authors	Diagnosis(es)	Research questions1	Inclusion criteria2	Search strategy3	Criteria for appraisal4	Process of critical appraisal5	Methods for minimizing error I data extraction6	Methods for combining studies7	Credibility8	Contribution to new knowledge of fatigue9
[20] Nielsen et al. 2019	MFS All types of articles on MFS	Good -	Good	Good	Not relevant	Good	Acceptable -	Good	Good	Good
[19] Velvin et al. 2014	MFS All types of articles on MFS	Good	Good	Good	Good	Good	Acceptable	Good	Good	Acceptable
[51] Velvin et al. 2019	HTAAD Only articles on MFS were identified	Good	Good	Good	Good	Good	Acceptable	Good	Good	Acceptable

*Recruitment and sample sizes:* In most papers [11–13, 16, 17, 31, 32, 52, 55, 58, 60–63, 65–70, 72–74] the participants were recruited from the clinic where the researchers worked, and in some [54, 56, 57, 59, 64] from the patient organization, or from both [53, 71]. Approximately, a total of 2,479 adults were included in the articles, with a variation of 16 [31] to 318 [54] respondents (mean 94/median 64), and in most studies the response rate was low. Four studies had more than 200 participants [54, 57, 63, 64], but the sample size of these studies probably represents a small percentage of the estimated national patient populations. In the largest study [54], the diagnosis was self-reported and the link to the survey was sent to 13,280 persons, of whom 318 completed the survey, indicating a response rate of 2% of the study population.

##### Instruments for assessing fatigue and vitality

Ten studies [11, 16, 17, 31, 63, 66, 67, 69, 71, 72] used Fatigue Severity Scale (FSS) two [55, 68] used Multidimensional Fatigue Inventory (MFI-20), two [32, 57] used Checklist Individual Strength (CIS), one [31] Fatigue Questionnaire and one [54] Quality of Life index. Eleven studies [13, 56–58, 62–65, 68, 71, 73] used SF-36 and one [52] used SF-12. Two studies [53, 64] used study specific questions to measure fatigue. Some studies [31, 57, 63, 64, 68, 71] combined two measurements. In studies of children, one study [70] used Patient Reported Outcomes Measurement Information System (PROMIS) and the other [61] Fatigue 10a Paediatric v20 short form. Table 4 shows an overview of the instruments used in the studies measuring fatigue and vitality.

**Table 4:** Instruments for assessing fatigue used in the different studies

Fatigue/vitality instruments	Study reference
*Adults*	
Fatigue Severity Scale (FSS)	[11, 16, 17, 31, 63, 66, 67, 69, 71, 72]
SF-36, subscale of vitality (SF-36,V)	[13, 56–58, 62–65, 68, 71, 73]
SF-12 (subscale of vitality (SF-12 V)	[52]
Multidimensional Fatigue Inventory (MFI-20)	[55, 68]
Fatigue Questionnaire(FQ)	[31]
Checklist Individual Strength subscale fatigue, pain (CIS)	[32, 57]
Quality of Life Index	[54]
Study specific (fatigue yes/no)	[53, 64]
*Children/adolescents*	
Patients Reported Outcome Measure (PROM-portal)	[70]
Fatigue 10a Paediatric v20 short form	[61]

*Control groups:* Four papers [32, 57, 58, 61] compared the results of fatigue with normative data and subgroups, three [7, 56, 65] with healthy controls matched for age and gender, three [13, 16, 17] with normative data and other diseases, six [31, 52, 53, 64, 68, 70] only with normative data. Two prospective studies [71, 73] included pre-/post results and normative data. Seven studies [11, 16, 54, 62, 63, 66, 67] did not describe any comparison group. There were large variations in the use of control groups and comparison to the general population and to other patient groups. None of the qualitative studies compared the results with normative data or other groups.

*Limitations and credibility:* Most studies (83%) had thoroughly described factors (confounders) that may negatively impact the credibility of the study, while three studies [13, 31, 62] had very limited description and two studies [64, 66] had no description of limitations. Omitting information about the study`s potential limitations may decrease the credibility of the study. The lack of credibility was also assessed related to other factors such as lack of verified diagnoses, small samples, limited transparency of the selection, methodology and analyzing process, the use of advanced statistical analyses in small sample sizes, no assessment of the validity and reliability of the measurements, low response rate and no drop-out or no non-response analyses. In addition, in qualitative studies, taking the role of the researcher into account and discussion on how researchers’ preconceptions may influence the results are important strategies for ensure trustworthiness and credibility of the study. (More detailed information about the assessments and justifications are available in Additional file 3).

Despite the included studies on fatigue in sHTADs being of limited in size, the overall quality of the methodology ranged from very good to fair, with most studies being rated as good. The credibility of a study combined with its results were used to decide how each study was assessed in contributing new knowledge about fatigue in sHTADs.

#### Synthesize and summary of results from included articles

**Fig. 2:**
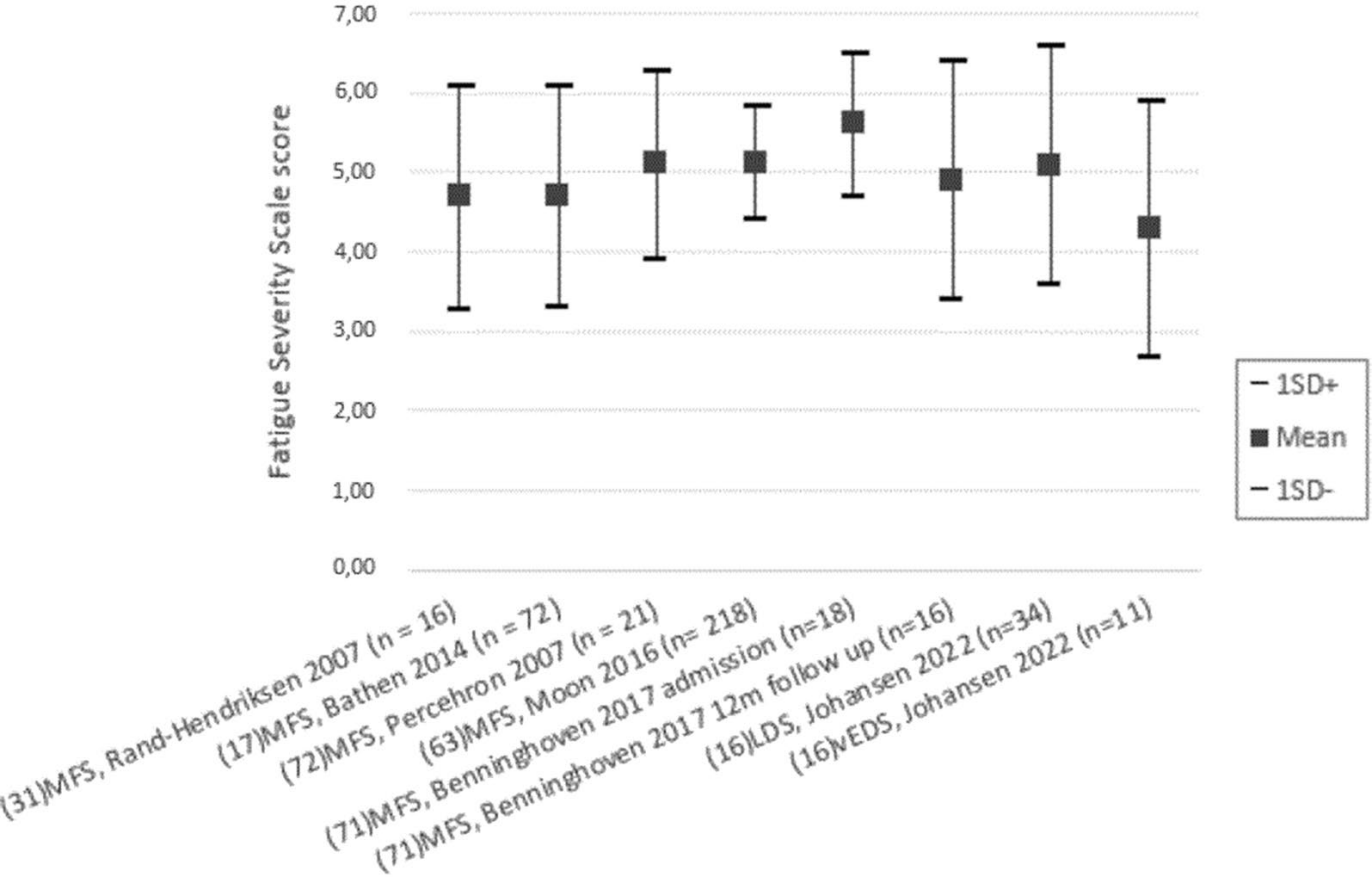
Mean Fatigue Severity Scale score in different sHTAADs

*Prevalence of fatigue and decreased vitality in adults with sHTADs:* Several studies [13, 16, 17, 32, 53, 55, 57, 58, 62, 64, 72, 73] presented increased fatigue (or decreased vitality) in adults with sHTADs compared to the general population, but the variation on prevalence of severe fatigue varied from 37% [32] to 88.5% [53] in MFS. Prevalence of severe fatigue in LDS was 58% [11, 16, 69] and 42% in vEDS [11, 16, 69], but these results were reported from the same study cohort. Comparing prevalence values was hampered by the fact that different instruments and cut of values were used for severe fatigue. Figure 2 shows the mean score of the different studies reported outcome on Fatigue Severity Scale (FSS). Four studies were not included in Fig. 2 because they reported results from the same study cohort as two studies illustrated in the figure. Higher fatigue scores signify more fatigue.

**Fig. 3:**
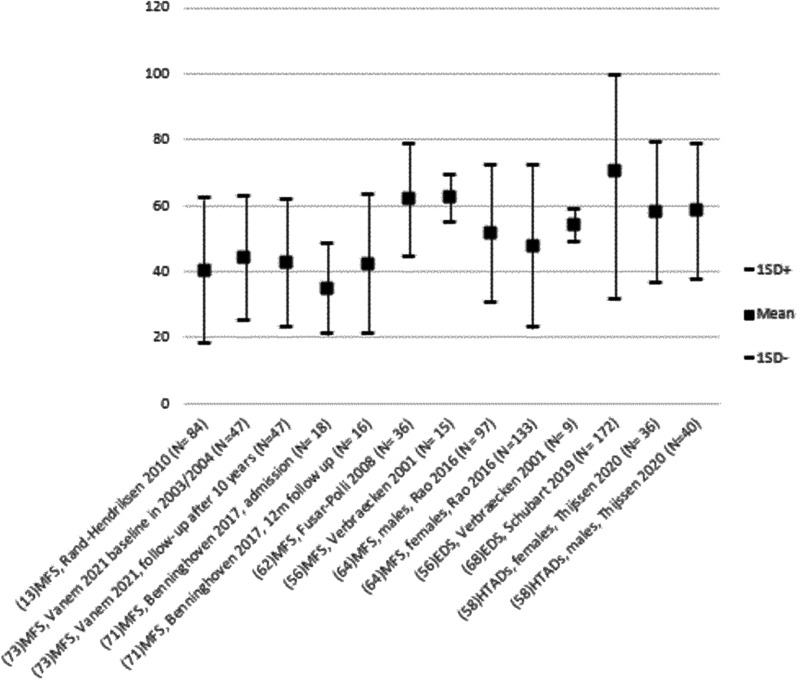
Mean SF-36 vitality score in the different sHTAADs

Prevalence of fatigue/reduced vitality was not reported in studies using SF-36. Figure 3 illustrate the mean score (+ 1SD) of the included studies reporting SF-36vt mean (SD). Very few studies indicated severe fatigue in adults with sHTADs according to SF-36vt scores of 35 and below [29], but results varied.

Some studies also compared their results to other diseases. Bathen et al. [17] found that adults with MFS had higher prevalence of fatigue than patients with rheumatoid arthritis, but lower than for other chronic conditions. Rand-Hendriksen et al. [13] found that the MFS study cohort scored lower of SF-36vt scores than all included comparison groups (hypertrophic cardiomyopathy, cystic fibrosis, Bechet’s syndrome). Rand-Hendriksen et al. [13] indicated that the low vitality score may reflect the common complaint of fatigue and reduced physical endurance among persons with MFS.

*Associated factors to fatigue in adults with sHTADs:* Many articles reported variables associated with fatigue and/or vitality. Fatigue was found to be positively associated with pain [16, 17, 32, 57], disability [31, 57, 61, 72], cardiovascular problems and multiorgan burden [16], sleeping problems or hyper somnolence [16, 56, 68], orthostatic intolerance [55], and psychological factors [16, 31, 32]. One study [53] indicated positive associations between fatigue and use of beta-blockers, while three other studies [17, 31, 55] found no such significant associations. One study [32] found that patients using losartan had less fatigue compared to those not using losartan. Factors associated with fatigue in sHTADs appear to be somewhat inconsistent, particularly about the association between medication and fatigue.

Fatigue was also found negatively associated to physical activity [11], work participation [17, 64, 66, 67, 69] and quality of life [54, 63, 65, 67, 69, 71], and positively associated to being female [31, 32, 58]. Only one qualitative study [12] was identified reporting some results on fatigue in adults with sHTADs. This study mainly focused on physical activity, but described that adults experienced it difficult to cope with fatigue in daily life. Fatigue and less physical activity seem to have a mutually reinforcing negative effect [12].

Several studies also reported other aspects of fatigue. Rao et al. [64] noted that fatigue was ranked as the third highest disease concern, while cardiac and spine problems were ranked first and second. This study also emphasized that MFS patients experienced specific difficulties in executive functions such as mental fatigue, leading to diminishing QoL. Severe fatigue seems to significantly heighten the perception of their condition`s severity, and experienced psychological distress may also increase fatigue [31, 32, 53, 54]. Interestingly, one pilot experimental intervention study [71] measured pre- and post-intervention fatigue and found that a three-week rehabilitation program significantly decreased the extent of fatigue in patients with MFS and LDS one year post-intervention. Several studies expressed the need for a specialized in-hospital rehabilitation program to deal with fatigue and other symptoms related to the disease.

*Fatigue in children with sHTADs:* The study samples of the five articles dealing with children and adolescents varied from 19 (74) to 147 (59) participants. Approximately, a total of 342 children and adolescents were included.

The results from the five studies [59–61, 70, 74] on children and adolescents with sHTADs are not consistent about the prevalence and extent of fatigue. One study [71] found that 44% of children with MFS reported fatigue sometimes or often. Another [61] found that children with MFS had less fatigue compared to the age matched norm data, and significantly lower prevalence of fatigue than children with hypermobile EDS. In the study [59] of adolescents with MFS on social media, fatigue was seldom not mentioned as a problem when discussing problems related to the diagnosis. Few studies had examined factors that are associated with fatigue in children with sHTADs, but one study [61] found that fatigue was significantly associated to disability and pain. Two qualitative studies [60, 74] reported that children and adolescents with MFS experience fatigue that limits school participation in sport and other activities. Children also described difficulties keeping up with their peers, feeling being different and therefore avoid social activities. This indicates that fatigue seems to be prevalent in children with sHTADs. However, the results were not consistent and more research is warranted.

### Results from the qualitative focus group interviews

#### Participants

A total of 36 individuals with sHTADs (14 with MFS, 11 with vEDS and 11 with LDS) participated in the focus group interviews. The participants represented a variety of diagnoses, gender, age, education and social backgrounds. The characteristics of the participants is shown in Table 5.

**Table 5:** Characteristics of the participants in focus groups

Demographic factors (n = 36)	
Age, mean (range) year	48 (22–71)
Women, n (%)	20 (56)
Educational level (highest finished education ≥ 13 years), n (%)	20 (56)
Working full time, n (%)	14 (39)
Working part-time combined with disability pension, n (%)	5 (13)
Full disability pension, n (%)	14 (39)
Students, n (%)	3 (9)
*Diagnoses*	
MFS, n (%)	14 (39)
LDS, n (%)	11 (30.5)
vEDS, n (%)	11 (30.5)

#### Themes and categories

Four main themes emerged from the analyses: (1) *Different diagnosis–different fatigue*? (2) *Nature of fatigue* (3) *Searching for causes of fatigue* (4) *Dealing with fatigue in daily life*. Overall fatigue was described as an overwhelming phenomenon, with an unpredictable character, and a feeling that fatigue dominated and controlled most life-situations. This is illustrated by the following quote:

“I really don’t know what to do, I never know and have no control, so for me fatigue is dominating my life” (an elderly woman with LDS).

1. *Different diagnosis—different experiences of fatigue?*

The focus groups were divided according to the diagnostic groups (2 groups with MFS, 2 groups with LDS and 2 groups with vEDS). No distinct differences emerged between the participants in the different disease groups on how they experienced and describes fatigue. They described that fatigue can be difficult to describe and explain, and many had rarely talked about it seriously before. Therefore, they emphasized that data collection with focus groups seemed to be constructive since it gave them opportunity to recall and confirm aspects with peers and share experiences. Most participants in all six focus groups reported that they experienced periodic or permanent fatigue, and there were no differences between the diseases. Despite the fact that having a sHTADs may be life threatening, many described fatigue as one of the most debilitating symptoms of the diagnosis. The following quote cover this on an overall level:

“I barely notice the severe symptoms of this diagnosis and sometimes I just forget it. But I get so tired of always being tired” (young man with vEDS).

2) *Nature of fatigue—how do they experience fatigue?*

Many of the participants experienced that fatigue was a combination of a bodily sensation and a foggy feeling. They described an overwhelming feeling of tiredness, and that parts of their body was heavy or paralyzed. Some described that their legs felt like lead, and they had trouble with moving. A misty feeling was described as having a glass bowl around their head, including brain fog. They felt it hard to concentrate, speak, listen or sort out impressions. Many described that it interfered with concentration at work and social interaction. The character of fatigue was also described as unpredictable, controlling their life, and being impossible to resist and combat.

“Yes, it’s weird, because I’m so incredibly tired. I can’t leave my bed, because my legs will not move, and my brain feels so foggy and I can’t think and talk right. This is not me… and I don’t know why” (woman with MFS).

3) *Searching for causes of fatigue*

Many expressed frustrations of neither knowing the cause of fatigue nor how to deal with an inexplicable exhaustion. Searching for biomedical and other reasonable reasons of fatigue was common. Some claimed that being hypermobile requires more body energy for stabilization of joint and muscles. Medication and use of blood pressure medicine was also mentioned as a possible explanation for fatigue. Others described how chronic pain and constant worrying about the life-threatening aspects of the condition for both them and family members made them exhausted. Many also described that being physically active and doing exercise was challenging, but despite that, exercise was emphasized as important in counteracting fatigue. Some mentioned also that physical activity was associated with anxiety and insecurity, due to the difficulties of finding the right balance between healthy and unhealthy activities. Avoiding physical activity was therefore a coping strategy for some, resulting in a negative spiral of inactivity, more fatigue and a sedentary life.

“I wish I had known the reason for my fatigue, it had been much easier to know what to do and combat it” (young man with LDS).

Another aspect many of the participants described as challenging was the suspicion and lack of understanding from other people about the phenomenon fatigue. When participants tried to convey the gravity of their experiences to others, they were not always understood. Participants described this as exhausting and it increased their uncertainty about the fatigue symptoms. This, in turn led to feeling of guilt, inadequacy and an inner struggle of their desires and what they could achieve.

“It is so invisible and common, everyone is exhausted sometimes… so people might understand in their own way… but they really don’t understand the reality of having “real fatigue” (middle aged woman with MFS).

4) *Dealing with fatigue in daily life*

Most of the participants described that fatigue impacted different aspects of daily life, such as family life, employment, and social life. Some emphasized that they had learned to cope with fatigue by energy economizing and prioritizing between different aspects of life. They realized that fatigue was not dangerous, but only frustrating and debilitating, and some tried to accept it as part of their illness. Others described that avoiding important life events because of exhaustion, was part of their daily choices. Difficulty of dealing with fatigue in the work situation was emphasized as a common challenge. With an overwhelming feeling of tiredness, it was impossible to fulfill their obligations as employees. For some, early retirement was the only solution. Others described attempts to strive to maintain full employment and trying to keep up the pace in what they perceived as a “normal life”. They used all their energy at work, which affected both family life and leisure activities. For some the long-term effect of keeping up the pace was increased fatigue because they were not able to rest enough. Others had found strategies for dealing with fatigue in the work life, particularly those who had informed their employer about their condition. More flexible work conditions and agreements with their employers, made it possible to maintain work ability, despite fatigue.

“For me, work is of invaluable value, I appreciate it so much, it gives me energy but it can also drain me of energy, so it is important to be aware and pay attention. Fatigue is so difficult to handle” (middle-aged man with MFS).

## Discussion

### Systematic review of relevant research of fatigue in patient with sHTADs

#### Identified research

Aim one was to identify, critically appraise and synthesize the research of self-perceived fatigue in sHTADs. We identified 33 articles dealing with fatigue in sHTADs, 3 reviews and 30 primary articles, but in only 8 articles, the primary outcome was to investigate fatigue. The results indicate that the amount and extent of studies on fatigue in sHTADs is very limited. Most articles included patients with MFS, presumably because MFS is more common and better known than the other diagnoses. The prevalence of these diseases worldwide is highly uncertain. Prevalence of MFS is estimated to be 10 per 100,000 [75, 76], and prevalence of vEDS is estimated to be 0.5–2 per 100,000 [77], while prevalence of LDS is unknown but probably much lower than for MFS [78]. The prevalence of the other sHTADs is even lower [5, 6]. Over the past two decades, there has been exponential increase in genetic research on pathogenic variants explaining sHTADs [1, 5, 6]. The increased focus on diagnostics, survival and treatment may have deflected clinical attention away from patient`s less dangerous symptoms, such as fatigue. LDS is a relatively new diagnosis, and first described in 2005 [4, 79]. Most studies of LDS concern medical aspects related to the diagnoses. Because most of the symptoms of MFS are overlapping with symptoms of LDS, vEDS and other sHTADs [4–6], it seems likely that fatigue impacts these patient groups similarly, although the pathophysiological mechanisms of fatigue may be different.

#### Critical appraisal of included articles

Most studies used quantitative cross-sectional questionnaire design, besides four prospective and four qualitative studies. The identified articles and results consistently were based on small sample sizes and/or low response rates. The respondents were mainly recruited from the clinic where the researchers worked or from patient organizations. These recruitment strategies yield a risk of bias for recruiting motivated persons with particular medical problems. Thus, the findings may be different from the non-respondents and therefore represent a bias related to the total population. Nearly all the patient populations lived in Western developed countries; Europe and USA or Korea. As cross-cultural differences of fatigue have been found in several studies [80], more research from other countries and cultures is warranted.

Nearly all quantitative questionnaire studies (cross-sectional and prospective) used standardized instruments with generic scale design, besides two papers using study specific questions about “fatigue being present or absent”. The use of standardized instruments can provide quantitative indication of fatigue level, but incompletely reported results, use of different instrument and use of different cut of values for severe fatigue made meta-analyses or statistical pooling difficult. Such meta-analyses could have been useful for clinical practice. The huge variations in prevalence of fatigue and vitality score within and between the different sHTADs may reflect differences in study design, recruitment routines, methodology, and national differences in cultures and perceptions. The representativeness and generalizability of the results may be questioned; however, a strength was that the methodology of included studies was commonly rated as good, and that most emphasized and discussed limitations of their study. This in turn, increased the credibility of the studies.

#### Synthesis of the literature

*The prevalence of fatigue:* The results from the reviewed papers are equivalent when indicating that adults with MFS have vastly higher prevalence of fatigue than the general population; and it is likely that individuals with sHTADs may experience a significant impact of fatigue on daily life. This is in accordance with reports of fatigue in other severe conditions such as systemic sclerosis [81], multiple sclerosis [82], and cancer [83].

Fatigue seems to be complex and can occur as primary or secondary symptom, as well as a comorbidity of an underlying disease [31, 68, 78]. Disentangling the origin and nature of fatigue in patients with sHTADs may be challenging. Several studies [13, 16, 31, 53, 55, 56] hypothesized that the biomedical aspects of sHTADs such as cardiovascular and respiratory factors, reduced visual acuity and joint hypermobility may impact fatigue, but only a few studies found such associations.

Fatigue has also been described as a well-recognized side-effect of blood pressure medicine [84], but the results from the included articles were contradictory. Only one [53] study found that medication was associated with more fatigue, while three studies found no such associations in patients with sHTADs. Interestingly the study of van Andel et al. [32] found that the use of losartan was associated with decreased fatigue. The effect of losartan on fatigue may be an under-researched aspect in these patient groups, and the limited and conflicting literature reveals that more research is needed. Our results also indicate that clinicians should be aware that fatigue may be exacerbated by the use of medication, and should enquire about the effects of medication on fatigue when assessing and prescribing new medications.

Chronic pain was also found to be significantly associated to fatigue in the included articles, similar to reports [85–87] on others chronic diseases. Pain in patients with sHTADs [14–16, 18] may contribute to lower energy level and lower sense of well-being [64]. One explanation is that chronic pain negatively influences sleep quality, and less sleep can decrease one`s pain threshold and pain tolerance as well as intensify the pain, thus exacerbating sleep problems, and thus increasing fatigue [87, 88].

Another aspect that emerged from the included articles was the close connection between physical activity and fatigue. Patients with sHTADs are often recommended physical restrictions because hemodynamic changes and increase in blood pressure are associated with an enhanced risk of aortic growth and acute aortic dissection [10, 88]. However, they are also recommended to be physical active. Total absence of physical exercise is deleterious and may lead to muscle wasting, joint stiffness, and problems with social and professional reintegration, depression and fatigue [10–12, 88]. Lack of exercise and deconditioning can cause tiredness and exhaustion. Finding the right balance between safe and unhealthy exercise for these patient groups can be a major problem [12]. Patients may experience anxiety related to physical activity, which in turn may lead to a sedentary lifestyle and more fatigue [9, 11, 12, 53, 89, 90], with these effects probably being negatively mutually reinforcing [91]. There are promising results that exercise-based cardiac rehabilitation in MFS and LDS can help these patients to decrease fatigue and chronic pain, with increased physical endurance and quality of life [71]. More clinical research, including more knowledge-based practice and rehabilitation guidelines for patients with sHTADs seems to be needed.

*Fatigue in children and adolescents:* Research on fatigue in children with sHTADs is very limited and the results varied. In three studies [60, 70, 74], fatigue was reported to create challenges in daily life, while in another [61] children with MFS reported less fatigue than healthy controls. One study [59] also indicated that fatigue was nearly not mentioned on social media when young people shared experiences about MFS. Nevertheless, more information about the prevalence, associations and experiences of fatigue in these pediatric groups is needed. Studies of children with other chronic diseases have revealed that fatigue negatively impact QoL [92], so quantifying fatigue in children with sHTADs may be critical. Understanding the impact of fatigue may be the first step for improving the quality of life for these groups.

### Focus group interviews combined with results from the review part

Aim two was to investigate the experiences and perceptions of fatigue in adults with different sHTADs. In the focus group interview, we did not measure the extent of fatigue, but our impression was that nearly all of the participants reported that they experienced fatigue. Participants in the focus group interviews emphasized that fatigue is one of the most debilitating symptoms of having a sHTAD, similar to the results from several of the studies from the review part. Fatigue seems to cause significant levels of distress, and the unpredictability and invisibility of the symptoms were emphasized as particularly challenging. Not being taken seriously and constantly being misunderstood was also highlighted as challenging and negatively affecting their self-esteem and self-understanding. Another challenge was to combine fatigue with the ability to work. Fatigue was reported to be the most prevalent reason for early retirement in the some of the included articles in the review [16, 17, 19, 66, 67].

Both in the review part and the focus groups it emerged that the subjective perception of the disease may have substantial impact on how people cope with fatigue [53, 64]. The physical severity of sHTADs has been discussed [13, 31], and in most papers, severity appears to be mainly associated with the cardiovascular manifestations [13, 53, 67]. The cardiovascular manifestations may be underestimated in both adults and children as long as the individuals experience no subjective complaints. The subjective severity seems to be mainly determined by the disease manifestations that is perceived by the patients or caused physical disability. The differences between physical severity and subjective severity may indicate that the patients perceive the disorder differently from the professionals. This is important for health care professionals to recognize when dealing with fatigue in these patient groups.

Overall, the results from our study indicate that the perception of fatigue is probably not an isolated problem, but rather a combination of factors related directly to the disease and psychological stress factors and indirectly to the lack of psychosocial support and the complex response of having a rare chronic disease. Based on these results, it is difficult to determine how widespread severe fatigue is among adults and children with sHTADs. Studies combining qualitative approaches with quantitative measurements such as FSS, MFI-20, CIS or other validated measurements could provide valuable information on both prevalence, associations and experiences of fatigue in these patient groups.

### Clinical implications and direction for further research

The third aim was to investigate key concepts of fatigue, identify knowledge gaps, and discuss clinical implications and direction for further research on fatigue in sHTADs.

The research on fatigue in the different sHTADs is limited, particularly in other sHTADs than MFS. Our results indicate that the concept of fatigue can be described in relation to its physical, cognitive, emotional and social impact. Further research in sHTADs can attempt to examine the concept of fatigue and unifying the taxonomy of discrimination between fatigue in sense of self-perception and performance (fatigability). This may help to clarify the complexity of the phenomenon. The negative consequences of fatigue seems to be consistent across sHTADs, as is the uncertainty concerning its underlying pathophysiological mechanisms. Therefore, more research about pathophysiologic mechanisms in sHTADs and other causes of fatigue are warranted.

Our results also indicate that it is important for health professionals to acknowledge and address the impact of fatigue on patients with sHTADs. The unpredictability of sHTAD related fatigue is dominant and pervasive, and is experienced as a vicious circle. Helping people to be able to understand and accept fatigue may be important to enable patients to manage and live with fatigue. Support from health professionals to manage fatigue and develop strategies to increase physical activity and maintain work is important for these patient groups.

Our review has shown that the use of many different fatigue measures and cut-off values, make comparisons across studies difficult. To overcome these challenges, our proposal is that multiple stakeholders like researchers, health professionals and patient organizations can cooperate to create standardized sets of outcomes relevant for the sHTADs. This will enable agreement on what aspects that are important to measure, how it should be measured and how results should be interpreted. International collaboration project for sHTADs may also be appropriate, including outcome measures for fatigue.

## Limitations and strengths

Only literature written in English, German, French and the Nordic languages were included in the systematic review, this might be a limitation. However, no studies written in other languages with English abstract were found. Our choice of search words and our cultural conceptual understanding may have limited our identification of papers and our interpretation of the content from the identified studies. We excluded case reports and studies with less than six participants. A strength may be the use of predefined criteria for critically appraising the literature, blinded by two reviewers and independently selected and categorized the studies, with the supervision of one other.

There are several limitations related to the focus group interviews. The retrospective perspective may imply possibility for recall bias. Our findings may also be limited to the patients with sHTADs who were willing to talk about their experiences and challenges. Another limitation may be how the term fatigue and exhaustion were interpreted by the participants. They may have different understanding of these concepts, but one of our intentions was also to examine the differences in perceptions and experiences about the concept of fatigue. Both the moderators and the co-moderators were experienced clinicians and/or researchers, and they underlined the interest in all types of narratives. In the analysis we carefully tried to identify and exclude repetitive patterns concerning our expectation and pre-understandings, as recommended in the literature [48, 50]. To ensure the transparency of the study, anonymized data are available on request to the authors (TRS, National resource Center).

## Conclusions

This is the first systematic review and qualitative study of fatigue in sHTADs. Our study indicate that fatigue is an under-recognized and under-researched feature in patients with sHTADs. A total of 33 articles were found, including several types of study designs. The majority dealt with MFS, and very few studies addressed other sHTADs. The studies were limited by small study sizes, low response rates, inadequate description of inclusion criteria and the patients’ diagnoses, and incomplete descriptions of the analyses. Despite these limitations, all studies indicated that the prevalence of severe fatigue in sHTADs is much higher than for the general population. The nature and impact of fatigue seems not to be experienced differently between the patients in the various sHTADs. Both the results from the included articles and the focus groups indicated that fatigue seems to have remarkable negative impact on daily life and quality of life. Fatigue may also be a major reason for early retirement. This suggests that fatigue should be considered as a core symptom and outcome measure in clinical trials and clinical practice for all patients with sHTADs. As most patients with sHTADs will not be cured in their lifetime, identifying causes of fatigue and developing appropriate treatment programs is warranted. Therefore, more research on fatigue in the different sHTADs are crucial.

## Supplementary Information

**Additional file 1.** The Prisma Checklist.

**Additional file 2.** The protocol of the study.

**Additional file 3.** Qualitity assessment of the included articles with justification.

**Additional file 4.** Steps of the inductive systematic text analysis

## Data Availability

The dataset supporting the systematic review part of the article is included within the article (and its Additional files). The dataset (transcribed interviews) from the qualitative focus group study is available on reasonable request to the corresponding author.
