# Supplementary material for: Fatigue in patients with syndromic heritable thoracic aortic disease: a systematic review of the literature and a qualitative study of patients’ experiences and perceptions
Source: Orphanet J Rare Dis. 2023 May 19;18:119. doi: 10.1186/s13023-023-02709-2 (PMC10199502; doi:10.1186/s13023-023-02709-2)
Supplement: Supplementary file 2 — Additional file 2. The protocol of the study. [file 13023_2023_2709_MOESM2_ESM.docx]

**Protocol for a systematic review planned published in 2023.**

**Title: Systematic review of Fatigue in persons with heritable thoracic aortic disease (HTAD); Marfan syndrome, Loeys Dietz syndrome, vascular Ehlers Danlos syndrome and other HTADs.**

Author information: Gry Velvin ¹, Heidi Johansen ¹, Amy Østertun Geirdal ², Trine Bathen¹

¹TRS National Resource Centre for Rare Disorders, Sunnaas Rehabilitation Hospital, Nesodden, Norway

² Department of Social Work, Child Welfare and Social Policy, Faculty of Social Sciences, Metropolian Univeristy of Oslo, Norway

Corresponding author: Gry Velvin, TRS National Resource Centre for Rare Disorders, Sunnaas Rehabilitation Hospital, Nesodden, Norway. ( e-mail: [gry.velvin@sunnaas.no](mailto:gry.velvin@sunnaas.no) )

**Abstract**:

**Background and scientific rationale for the study**

We present a protocol for a systematic review of current literature on fatigue in persons with a Hereditable Thoracic Aortic Disease (HTADs), such as Marfan syndrome (MFS), Loeys Dietz syndrome (LDS), vascular Ehlers Danlos syndrome (vEDS) and other HTADs. We are not aware of previous systematic reviews addressing aspects of fatigue in HTADs. A systematic review with an overview of relevant published papers on fatigue in HTADs could serve as a baseline for further studies including an overview of the characteristics of investigated patient populations, different research questions and the methods used to investigate fatigue. A systematic review may also provide a better understanding of fatigue-related aspects and can provide important information for developing more knowledge-based practice for people with HTADs.

**The aims**

The aims of our systematic review are (i) to identify publications of fatigue in patients with HTADs, (ii) to critically appraise the identified literature, (iii) and analyze and synthesize what the research reveals about fatigue in these patient groups, (iv) discuss clinical implications and directions for future research on fatigue in HTADs.

**Method**

A systematic review will be conducted by searching the published literature in all relevant databases and other sources. Studies will be selected based on predefined inclusion criteria and will include all types of primary studies and reviews. A flow chart will detail the flow of papers through the different stages of the review, including reasons for exclusion and inclusion. Each paper will be critically appraised using specific validated criteria for different study designs and presented in tables with explanations and justifications of the quality assessment of included studies. The synthesizing of results will be charted and presented in tables, including; reference details, design, methods and measurements, results related to fatigue finding (such as prevalence, association, experiences, and intervention outcomes). The results will give an overview of available research evidence, indicate the quality of the papers and the relevance for clinical practice, identify knowledge gaps, and serve as a basis for making recommendations for future research.

Keywords: Heritable Thoracic Aortic Disease, Marfan syndrome, Loeys-Dietz syndrome, vascular Ehlers- Danlos syndrome, systematic review, fatigue, vitality, exhaustion, dizziness, vitality.

1. **Introduction and the scientific rationale for the study HTAD**
   1. **Introduction**

This is a protocol for a systematic review study of fatigue in patients with heritable thoracic aortic diseases (HTADs). According to systematic review methodology, the preparation of a protocol is an essential component of the systematic review process to promote transparency and consistency through the review process, and to prevent selection bias (1,2,3). HTADs are a group of genetic conditions affecting the aorta and other major arteries (4,5,6,7,8). An extensive list of human genes and clinical implications associated with HTADs are described in several papers (7,8). According to Renard (8) approximately 53 genes are associated with HTADs, but only 11 genes (COL3A1, FBN1, SMAD3, TGFB2, TGFBR1, TGFBR2, ACTA2, MYH11, MYLK, LOX and PRKG1) were assessed as having a “definitive” and “strong” gene-disease association during the curation process (8). Mutations in the five first genes are defined as non syndromic HTAD (nsHTAD), with vascular manifestation alone (4,8,9). The most common syndromic HTADs are Marfan syndrome (MFS), Loeys-Dietz syndrome (LDS) and vascular Ehlers-Danlos syndrome (vEDS). These diagnoses show clinical overlap regarding cardiovascular, skeletal, craniofacial, ocular and cutaneous features (7,8,10,11). The most serious complications in HTADs are related to the cardiovascular system with risk of aneurysm and dissection of aorta and other large arteries (4). Life-threatening complications can require emergency intervention without warning, with increased risk of subsequent morbidity and potential loss of physical function (4,11). Many have skeletal sign with hypermobile joints, chest deformities and scoliosis (6,7). Because of the risk of aortic dissection, many patients are advised to refrain from contact sport, to limit physical exertion and strict blood pressure control (12,13). Unfortunately, for some followed by inactivity and a sedentary lifestyle (13,14,15,16). Physical impairment, fatigue and chronic pain may be exacerbated by the fact that most HTADs have no effective treatment or cure, and the research is limited (4,6,17,18,19,20,21). The experience of living with a HTAD disease is vastly more complex than its medical features (16,21,22). Any aspects of life may be affected, but there is a lack of clarity as what actually contributes to fatigue in patients with HTADs. It seems that fatigue is an under-recognized and under-researched aspect in most HTADs. Both more clinical understanding of the mechanisms of fatigue in HTADs and more research seem warranted.

- 1. ***Fatigue***

In recent years fatigue has increasingly been recognized as an important clinical factor affecting several aspects of patients` lives. A problem is that fatigue is a complex phenomenon lacking a clear and unanimous or overarching theory. The term fatigue refers to a variety of related conceptually distinct constructs. Authors often do not explicit define fatigue, but rather imply its meaning by the constructs they measure (23,24,25). Other similar terms used in the literature of fatigue is “lack of vitality”, “exhaustion”, “tiredness”, “mental or/and physical fatigue”. (27,29). One common definition is “an overwhelming sense of tiredness, lack of energy and feeling of exhaustion, mental, physical or both (27,29). Several attempts have been tried to classify fatigue according to affected domains (motor versus cognitive) or it presumptive origin (central versus peripheral) (25). However, these definitions do not take into account that subjective perception of fatigue and severity deficits in motor or cognitive tasks (fatigability) often are not identifiable. In our study the main focus is on self-perceived fatigue.

There is also a wealth of distinct and discrepant scales created to measure fatigue, both generic and disease-specific (25,29). Disease-specific measures developed specific to assess these HTADs may help obtain more accurate information about how HTAD diagnoses affect fatigue. As far as we know, no such measurements have been developed.

Vitality has in several studies been described the opposite of fatigue, low degree of vitality may indicate severe fatigue. The Rand version of SF-36 Health Survey (30) is a health quality of life measure with the subscale of vitality defined as a scale for measuring general energy, lack of vitality i.e. similar to fatigue (26,28,31). Vitality is found strongly to correlate with different validated measures of fatigue (26,28,31). Therefore, in our review we find it appropriate to include the vitality scores from SF-36.

- 1. **Fatigue in HTADs**

Fatigue is a common feature in different diseases, and may have multiple contributing factors (e.g., sleep disorders, pain, reduced physical activity, depression and pharmacotherapy (32). There might be multiple association or contributing factors associated to fatigue in people with HTADs e.g. sleep disorder, pain, reduced muscle activity, cardiovascular and respiratory factors, working capacity, use of beta-blockers, reduced visual acuity, scoliosis, joint hypermobility, cognitive function, psychological distress and demographic aspects (e.g. age, gender, work participation, education, having children, living along or married).

Figure 1 described how the complexity of the diagnoses and daily life aspects might influence fatigue in patients with HTADs.

***Figure 1: HTAAD interaction between health-realted symptoms, daily life and fatigue***


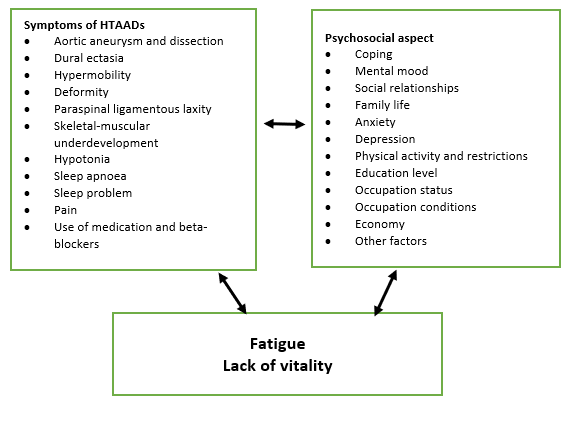


1. **The rationality for the systematic review**

There is a need for more research on fatigue in HTADs. A systematic overview of fatigue measurements, assessment quality and findings in patients with HTADs will be conducted.

Systematic reviews are viewed as essential for both healthcare providers and policy-makers to guide clinical practice and develop trustworthy clinical guidelines. Systematic reviews are also essential to establish what is already known and what is needed in further research (33). A systematic review of fatigue in HTADs may be of importance for clinical practice, further research and as a basis for evaluating the need for developing disease specific scales related to potential direct effects of the particular condition.

A systematic review could serve as a baseline for further studies, the amount of primary research studies versus secondary studies (systematic reviews) and if fatigue related aspects are primary outcome or not. An overview of the characteristics of investigated patient populations, different research questions and the methods used to investigate fatigue in HTADs may provide a better understanding of the prevalence, associations, and how people experience fatigue. Further, the results will provide an overview of available research evidence, indicate the quality of the papers and the relevance for clinical practice, identify knowledge gaps, and serve as a basis for making recommendations for future research. The review may also generate knowledge that can be important for developing more knowledge- based practice and for developing guidelines for monitoring these patient groups.

1. **Objectives and research questions**
   1. ***Objectives***

A systematic review of relevant research on fatigue in patients with a HTAD diagnoses will be conducted. The objective is fourfold; (i) to identify publications of fatigue in patients with HTADs, (ii) to critically appraise the found literature, (iii) analyze and synthesize what the research reveals about fatigue in these patient groups (iv) and discuss clinical implications and directions for future research on fatigue in HTADs.

- 1. ***The research questions:***
- What is the extent and characteristics of the studies on fatigue in HTADs (e.g., MFS, LDS, vEDS)?
- How much focus is given to experienced fatigue in the studies? (i.e. experienced fatigue primary or secondary aim/outcome),
- What are the characteristics of study populations? (i.e. types of diseases, sample sizes, adults/children), and when and where have these studies been carried out? (i.e. publication year and country of participants).
- What types of study designs have been used (e.g., quantitative cross sectional studies, qualitative, retrospective, RCT etc).
- What types of assessment methods have been used to investigate experienced fatigue in persons with rare diseases? (i.e. study-specific or standardized fatigue questionnaires and qualitative interview methods)
- What do the results of the studies on fatigue in HTADs indicate (prevalence, predictors/associations, experiences, perceptions, outcomes etc.)?
- How is the methodological quality of these studies?
- Which predictors/associations are identified in association to fatigue in HTADs?
- Do the results from included studies have any implication for clinical practice or futher research?

1. **Method**
   1. ***Study design***

Systematic review will be conducted using the recommendations for systematic reviews [33,34, 35,36] and quality assessment for different types of studies [37,39,39]. Due to limited amount of studies, all types of studies will be included, quantitative, qualitative primary studies and review studies. This will gain an overview of relevant research on fatigue.

- 1. ***Inclusion and exclusion criteria***

The criteria comprise basic inclusion criteria as well as quality criteria. The criteria were developed based on a preliminary review of a random subset of relevant fatigue studies and informed by theoretical literature on the different conceptual understanding of fatigue (21). A table will be prepared to concretize and visualize the inclusion and exclusion criteria.

*Three basis selection criteria are developed:*

1) Individuals affected with a specific HTADs. No exclusions will be made on the basis of age, gender or ethnicity. Studies with mixed populations including HTADs diagnoses that did not report subgroup analyses will be excluded.

2) All types of peer-reviewed studies presenting own results, published in English, German, French or Nordic language will be included. Unpublished data, short communications, clinical guidelines, letters to editor, expert statement, opinion, poster presentations and case-report studies with less than four participants will be excluded.

3) The third inclusion criteria is related to the aim/outcome of the study “at least one primary aim was to describe fatigue and/or predictor variables or factors associated with fatigue in HTADs”. We also will included articles using vitality score, because this is defined as the opposite as fatigue (Brown 26, Penner 25, McCabe 27). The lower vitality score on e.g. SF-36, the more fatigue.

- 1. ***Literature search:***

Systematic searches strategies will be conducted in the following databases: PubMed, MEDLINE, Cochrane, Embase, Cinahl, Eric, Google Scholar and Web of Science (literature from 1990 to October 2022). The following search terms and Boolean operators will be used: Heritable thoracic aortic aneurysm and dissection OR *heritable* aortic disease OR hereditary thoracic aortic diseases OR familiar thoracic aortic aneurysm dissection OR genetic aortic diseases FTAAD OR FTAD OR HTAAD OR HTAD OR TAD OR Genetic Aortic Disease AND Marfan syndrome OR Loeys-Dietz OR vascular Ehlers-Danlos OR Ehlers Danlos syndrome Or Rienhoff OR arterial tortuosity OR familial thoracic aortic OR Heritable thoracic aortic OR aneurysm osteoarthritis syndrome OR FTAAD OR HTAAD OR TAAD OR ACTA2 OR MYH11 OR FBN1 OR TGFBR1 OR TGFBR2 OR TGFB2 OR TGFB3 OR SMAD3 OR MYLK OR PRKIG1 OR COL3A, OR LOX OR PRKG1, and all the genes associated with thoracic aortic aneurysm and dissection (7,8). Another search will be done in the same databases with the following terms: Fatigue OR exhaustion OR dizziness OR sleep problems OR sleep apnea OR vitality OR dizziness. Then search one will be (AND) combined with search two. References in included articles both the review and primary papers will be examined. In addition, searches in grey literature will be conducted. Leading experts in the field HTADs (people with excellent clinical experiences and professionals with several publications on fatigue, pain, QoL and clinical aspects and psychosocial aspects in peer reviewed scientific papers on HTADs) will be consulted for additional literature.

- 1. ***Review and data extraction:***

Two researchers (authors) will independently review the abstract and/or articles from each citation that will be identified through the search strategy described above. When considered potentially eligible, the complete text of these studies will be obtained and reviewed by the same two researchers against the eligibility criteria to determine their eligibility. Then the third and fourth researchers will verify the article inclusions or exclusions and do the final eligibility. All included articles will be screened and categorized independently by two reviewers on the basis of the content in the articles [1,2,33,34,35). Each paper will be critically appraised by using specific validated criteria for different types of studies (36,37,38), by the same two reviewers. Two other reviewers will assess the results to ensure accuracy and the quality of the critical appraisal.

A priori data extraction and synthesizing will be conducted (1,2,3,33) of two reviewers by collecting information on the following reference: (title, author and publication year, study population, diagnostic information, recruitment source, country, study design and methodology; fatigue measurements and vitality sub scores measurement, key predictor variables, intervention and comparator (if applicable), key findings, authors conclusion and implication for clinical practice, study limitations and study funding). Then two other researchers will make assessment of the preliminary data extraction and synthesizing to ensure the accuracy and comprehensiveness of the results included. Discrepancies and disagreement will be discussed and resolved by involving a fourth researcher. The findings from the different studies will be synthesized and categorized in specific themes described in the paper.

- 1. **Collating, appraising, summarizing, and reporting of results*:***

A flow chart will present the information through the different stages of the review, included and excluded references, and reasons for exclusion, and number of papers on each disease. The critical appraisal will be presented in a table describing the quality assessment based on the specific criteria (36,37,38) for the different studies included in our review. A matrix will be used for summarizing and synthesizing the results from the different studies. Issues of special interest will be: statistical comparison with healthy controls/population norms or other diseases; analysis of associations with related health problems and fatigue predictors. The outcome and estimate will be presented as described in the original papers. We will probably not be able to perform any statistical procedures or meta-analysis due to small sample sizes and the heterogeneity of studies. We will probably rather use a narrative synthesis of the findings taking in account methodological quality and analytic rigor in the examination and reporting of findings.

- 1. **Evaluation and discussion of the findings of the systematic review:**

The results will provide an overview of the available research on fatigue in patients with HTADs and identify research gaps. We will discuss the quality of the instrument/ measurements used in the different studies and disadvantages/advantages of using these different measurements in groups with HTADs. The discussion will also deal with how different variables may be associated to fatigue in HTADs and in which way the available evidence may be relevant for clinical practice, documentation for developing clinical guidelines and preventive strategies and recommendations for future research.

1. **Conclusion:**

We consider a systematic review to be a useful method for quality assessment and synthesizing research within fatigue in HTADs. The findings could reveal gaps in the current research; serve as a source for future research; identify risk of bias assessments and judging the quality of the evidence. The method and data will be presented transparent, in a manner that provide the reader with useful information for an overall judging of the present research evidence on fatigue in persons with HTAD diagnoses.

**Referanser**

1. Cambell collaboration: Cambell collaboration of systematic review: [What is a systematic review? - The Campbell Collaboration](https://www.campbellcollaboration.org/what-is-a-systematic-review.html)
2. The Cochrane Handbook for systematic reviews: [Cochrane Handbook for Systematic Reviews of Interventions | Cochrane Training](https://training.cochrane.org/handbook) –
3. PRISMA 2020 Checklist for Systematic Review: [Microsoft Word - PRISMA 2009 Checklist.doc (prisma-statement.org)](http://www.prisma-statement.org/documents/PRISMA_2020_checklist.pdf)
4. Isselbacher EM, Preventza O, Black HJ, Augoustides JG, Beck AW, Bolen MA et al. Guideline for the Diagnosis and Management of Aortic Disease: A Report of the American Heart Association/American College of Cardiology Joint Committee on Clinical Practice Guidelines. Circulation. 2022;2. DOI: [10.1161/CIR.0000000000001106](https://doi.org/10.1161/cir.0000000000001106)
5. Landstrom AP, Kim JJ, Gelb BD, Helm BM, Kannankeril PJ, Semsarian C et al. American Heart Association Council on Genomic and Precision Medicine; Council on Lifelong Congenital Heart Disease and Heart Health in the Young; Council on Arteriosclerosis, Thrombosis and Vascular Biology; and Council on Lifestyle and Cardiometabolic Health. Genetic Testing for Heritable Cardiovascular Diseases in Pediatric Patients: A Scientific Statement From the American Heart Association. Circ Genom Precis Med. 2021;14(5). DOI: [10.1161/HCG.0000000000000086](https://doi.org/10.1161/hcg.0000000000000086)
6. Meester J, Verstraeten A, Schepers D, Alaerts M, Van Laer L, Loeys BL. Differences in manifestations of Marfan syndrome, Ehlers-Danlos syndrome, and Loeys-Dietz syndrome. Ann Cardiothorac Surg. 2017;6(6):582-594. doi:10.21037/acs.2017.11.03. PMID: 29270370; PMCID: PMC5721110.
7. Brownstein AJ, Ziganshin BA, Kuivaniemi H, Body SC, Bale AE, Elefteriades JA. Genes Associated with Thoracic Aortic Aneurysm and Dissection: An Update and Clinical Implications. Aorta (Stamford). 2017;1;5(1):11-20. doi: 10.12945/j.aorta.2017.17.003. PMID: 28868310; PMCID: PMC5570562.
8. Renard M, Francis C, Ghosh R, Scott AF, Witmer PD, Adès LC et al. Clinical Validity of Genes for Heritable Thoracic Aortic Aneurysm and Dissection. J Am Coll Cardiol. 2018;72(6):605-615. DOI: [10.1016/j.jacc.2018.04.089](https://doi.org/10.1016/j.jacc.2018.04.089)
9. Monda E, Lioncino M, Verrillo F, Rubino M, Caiazza M, Mauriello A et al. The Role of Genetic Testing in Patients with Heritable Thoracic Aortic Diseases. Diagnostics (Basel). 2023;17;13(4):772. DOI: [10.3390/diagnostics13040772](https://doi.org/10.3390/diagnostics13040772)
10. Papatheodorou E, Degiannis D, Anastasakis A. Genetics of Heritable Thoracic Aortic Disease (Review). Cardiogenetics 2022;12, 63–79. https://doi.org/10.3390/ cardiogenetics12010006
11. Bradley TJ, Bowdin SC, Morel CF, Pyeritz RE. The Expanding Clinical Spectrum of Extra cardiovascular and Cardiovascular Manifestations of Heritable Thoracic Aortic Aneurysm and Dissection. Can J Cardiol. 2016;32(1):86-99. doi:10.1016/j.cjca.2015.11.007.
12. Jensen TL, Tran P, Kjaer M. Marfan Syndrome and Exercise: A literature review. Trans Sport Med. 2020;3(6):526-535. [doi.org/10.1002/tsm2.185](https://doi.org/10.1002/tsm2.185)
13. Thijssen CGE, Bons LR, Gökalp AL, Van Kimmenade RRJ, Mokhles MM, Pelliccia A et al. Exercise and sports participation in patients with thoracic aortic disease: a review. Expert Rev Cardiovasc Ther. 2019,17(4):251-266. DOI: [10.1080/14779072.2019.1585807](https://doi.org/10.1080/14779072.2019.1585807)
14. Johansen H, Velvin G, Lidal I. Adults with Loeys-Dietz syndrome and vascular Ehlers-Danlos syndrome: a cross-sectional study of patient experiences with physical activity. Disabil Rehabil. 2020;1-8. [doi.org/10.1002/ajmg.a.61396](https://d.docs.live.net/ce4f393f79188249/From%20DB/Reviewer/OJRD-D-22-00832%20Hereditable%20thoracic/doi.org/10.1002/ajmg.a.61396)
15. Velvin G, Wilhelmsen JE, Johansen H, Vardeberg K, Lidal I. Physical exercise for people with hereditable thoracic aortic disease. A study of patient perspectives. Disabil Rehabil. 2019:1-8. [doi.org/10.1080/09638288.2019.1703145](https://d.docs.live.net/ce4f393f79188249/From%20DB/Reviewer/OJRD-D-22-00832%20Hereditable%20thoracic/doi.org/10.1080/09638288.2019.1703145)
16. Rand-Hendriksen S, Johansen H, Semb SO, Geiran O, Stanghelle JK, Finset A. Health-related quality of life in Marfan syndrome: A cross-sectional study of Short Form 36 in 84 adults with a verified diagnosis. Genet Med. 2010;12(8):517-524. [gim201083.pdf (nature.com)](https://www.nature.com/articles/gim201083.pdf)
17. Velvin G, Bathen T, Rand-Hendriksen S, Geirdal AØ. Systematic review of chronic pain in persons with Marfan syndrome. Clin Genet. 2016;89(6):647-658.   DOI: [10.1111/cge.12699](https://doi.org/10.1111/cge.12699)
18. Johansen H, Velvin G, Lidal IB. Pain and fatigue in adults with Loeys-Dietz syndrome and vascular Ehlers-Danlos syndrome, a questionnaire-based study. Am J Med Genet A. 2022;188(9):2605-2616. DOI: [10.1002/ajmg.a.62858](https://doi.org/10.1002/ajmg.a.62858)
19. Bathen T, Velvin G, Rand-Hendriksen S, Robinson HS. Fatigue in adults with Marfan syndrome, occurrence and associations to pain and other factors. Am J Med Genet A. 2014;164A(8):1931-9. DOI: [10.1002/ajmg.a.36574](https://doi.org/10.1002/ajmg.a.36574)
20. Nelson AM, Walega DR, McCarthy RJ. The Incidence and Severity of Physical Pain Symptoms in Marfan Syndrome: A Survey of 993 Patients. Clin J Pain. 2015;31(12):1080-6. DOI: [10.1097/AJP.0000000000000202](https://doi.org/10.1097/ajp.0000000000000202)
21. Velvin G, Bathen T, Rand-Hendriksen S, Geirdal AØ. Systematic review of the psychosocial aspects of living with Marfan syndrome. Clin Genet. 2015;87(2):109-16. DOI: [10.1111/cge.12422](https://doi.org/10.1111/cge.12422)
22. Rand-Hendriksen S, Sørensen I, Holmström H, Andersson S, Finset A. Fatigue, cognitive functioning and psychological distress in Marfan syndrome, a pilot study. Psychol Health Med. 2007;12(3):305-13. DOI: [10.1080/13548500600580824](https://doi.org/10.1080/13548500600580824)
23. Kluger BM, Krupp LB, Enoka RM. Fatigue and fatigability in neurologic illnesses: proposal for a unified taxonomy. Neurology. 2013;80(4):409-416 DOI: [10.1212/WNL.0b013e31827f07be](https://doi.org/10.1212/wnl.0b013e31827f07be)
24. Dittner AJ, Wessely SC, Brown RG. The assessment of fatigue: a practical guide for clinicians and researchers. J Psychosom Res. 2004;56(2):157-170.DOI: [10.1016/S0022-3999(03)00371-4](https://doi.org/10.1016/s0022-3999(03)00371-4)
25. Penner IK, Paul F. Fatigue as a symptom or comorbidity of neurological diseases. Nat Rev Neurol. 2017;13(11):662-675. DOI: [10.1038/nrneurol.2017.117](https://doi.org/10.1038/nrneurol.2017.117)
26. Brown LF, Kroenke K, Theobald DE, Wu J. Comparison of SF-36 vitality scale and Fatigue Symptom Inventory in assessing cancer-related fatigue. Support Care Cancer. 2011 Aug;19(8):1255-1259. doi: 10.1007/s00520-011-1148-2.
27. Maher C, Crettenden A, Evans K, Thiessen M, Toohey M, Watson A et al. Fatigue is a major issue for children and adolescents with physical disabilities. Developmental Medicine & Child Neurology. 2015. DOI: [10.1111/dmcn.12736](https://doi.org/10.1111/dmcn.12736)
28. McCabe M. Fatigue in children with long-term conditions: an evolutionary concept analysis. Journal of Advanced Nursing. 2009;65(8):1735-1745. DOI: [10.1111/j.1365-2648.2009.05046.x](https://doi.org/10.1111/j.1365-2648.2009.05046.x)
29. Bathen T, Johansen H, Strømme H, Velvin G. Experienced fatigue in people with rare disorders: a scoping review on characteristics of existing research. Orphanet J Rare Dis. 2022;10;17(1):14. DOI: [10.1186/s13023-021-02169-6](https://doi.org/10.1186/s13023-021-02169-6)
30. 36 RAND corporation ([36-Item Short Form Survey (SF-36) Scoring Instructions | RAND](https://www.rand.org/health-care/surveys_tools/mos/36-item-short-form/scoring.html)
31. Overman CL, Kool MB, Da Silva JA, Geenen R. The prevalence of severe fatigue in rheumatic diseases: an international study. Clin Rheumatol. 2016;35(2):409-15. DOI: [10.1007/s10067-015-3035-6](https://doi.org/10.1007/s10067-015-3035-6)
32. Dittner AJ, Wessely SC, Brown RG. The assessment of fatigue: a practical guide for clinicians and researchers. J Psychosom Res. 2004;56(2):157-170.DOI: [10.1016/S0022-3999(03)00371-4](https://doi.org/10.1016/s0022-3999(03)00371-4)
33. Pluye P. Critical appraisal tools for assessing the methodological quality of qualitative, quantitative and mixed methods studies included in systematic mixed studies reviews. J Eval Clin Pract. 2013;19(4):722. doi: 10.1111/jep.12017.
34. Fowkes FG, Fulton PM. Critical appraisal of published research: introductory guidelines. BMJ. 1991;11;302(6785):1136-1140. doi: 10.1136/bmj.302.6785.
35. Greenhalgh T. Assessing the methodological quality of published papers. BMJ. 1997; 2;315(7103):305-308. doi:10.1136/bmj.315.7103.305.
36. Jack L Jr, Hayes SC, Scharalda JG, Stetson B, Jones-Jack NH, Valliere M et al. Appraising quantitative research in health education: guidelines for public health educators. Health Promot Pract. 2010;11(2):161-165. doi: 10.1177/1524839909353023.
37. Ryan F, Coughlan M, Cronin P. Step-by-step guide to critiquing research. Part 2: Qualitative research. Br J Nurs. 2007;16(12):738-744. DOI: [10.12968/bjon.2007.16.12.23726](https://doi.org/10.12968/bjon.2007.16.12.23726)
38. Jeanfreau SG, Jack L Jr. Appraising qualitative research in health education: guidelines for public health educators. Health Promot Pract. 2010;11(5):612-617. DOI: [10.1177/1524839910363537](https://doi.org/10.1177/1524839910363537)
