# Supplementary material for: Fatigue in patients with syndromic heritable thoracic aortic disease: a systematic review of the literature and a qualitative study of patients’ experiences and perceptions
Source: Orphanet J Rare Dis. 2023 May 19;18:119. doi: 10.1186/s13023-023-02709-2 (PMC10199502; doi:10.1186/s13023-023-02709-2)
Supplement: Supplementary file 3 — Additional file 3. Qualitity assessment of the included articles with justification. [file 13023_2023_2709_MOESM3_ESM.docx]

| **Table 4. Quality assessment of included studies:**  **Quantitative studies, qualitative studies and review studies** | | | | | | | | | | | | | | | | | | | | |
| --- | --- | --- | --- | --- | --- | --- | --- | --- | --- | --- | --- | --- | --- | --- | --- | --- | --- | --- | --- | --- |
| **Quantitative studies** | | | | | | | | | | | | | | | | | | | | |
| **Quality assessment criteria**  Is the study design identified and appropriate?  How representative are the study group for the population?  Is there adequate control group?  Is the validity for measurement acceptable?  Is the study complete with regard to dropout/missing data and reporting respond rate?  Do the authors describe and discuss limitations with the study?  To what extent are study results influenced by factors that negatively impact their credibility?  Does the study contribute to (new) knowledge about fatigue in sHTAD? | | | | | | | | | | Ratings: Very good, Good, Acceptable, Fair and Poor | | | | | | | | | | |
| **Authors**  **Years** | | **sHTAD diagnosis** | | **Study design** 1 | | **Representative sample** 2 | | **Control groups** 3 | | **Fatigue measure validity** 4 | | **Dropout/ missing data etc. 5** | | **Discuss limitations** 6 | | **Credibility** 7 | | | **Novel knowledge about fatigue** 8 | |
| **17**  Bathen et al 2014 | | MFS:  All respondents  had verified Ghent 1 | | **Good**  -Clearly described and design seems reasonable for the purpose of the study.  -Adequate statistical analyses for small sample sizes. | | **Good**  -Small sample size, but verified diagnosis  -May represent a large amount of the patient population in the country.  -Gender approximately similar and good age variation. | | **Good**  -Compared fatigue to norm data and two other diseases, published data.  -Not matched control group**.** | | **Good**  **-** Generic standardized instrument (FSS)  -Discuss the instruments general validity and reliability.  -Present cut-off value for fatigue.  - Discuss the suitability for the study group | | **Good**  -Recruitment process, Inclusion/exclusion, response rate, non-respondents, and missing data reported.  -Drop-out not reported. | | **Good**  -Description of limitation.  About the representativeness of the study sample and the use of self-constructed questionnaire  (separate section) | | **Good**  -All verified diagnosis, appropriate study design and methods, but small sample size, use of standardized instruments and, transparent description of recruitment process the statistical analysis and limitations. | | | **Very good**  -First relatively large study with main focus of fatigue in MFS  -New knowledge about prevalence of fatigue  -Gives reasonable explanation about variables associated to fatigue  -Described implication for clinical practice | |
| **71**  Benninghoven et al 2017 | | MFS (one with LDS:  All with MFS verified Ghent 1/Ghent 2 | | **Good**  -The design clearly described and well-reasoned  -Design and methods seems appropriate for the purpose of the study.  -Procedures and intervention thoroughly described  -Statistical analyses based on very small sample | | **Acceptable**  -Very small sample size, but verified diagnosis.  - Presumably not representative for the MFS population in the country.  -Skewed gender distribution (71% female), age distribution not described. | | **Good**  Compare pre-and post-intervention, general population and norm data | | **Good**  - Generic fatigue instrument (FSS) and separate data for SF-36 vitality score  - Discuss briefly the instruments‘ general validity and reliability  -Nothing about suitability for the patient groups. | | **Good**  –Recruitment process, inclusion/exclusion criteria, missing data and drop-out described.  -Response rate not described (not relevant). | | **Very good**  - Limitations comprehensively described about small study-sample, unclear direction of causality etc. These were tried ameliorated thoroughly discussed (separate section) | | **Good**  -All verified diagnosis, appropriate study design and methods, but small sample size, use of standardized instruments and, transparent description of recruitment process, limitation and pre/post statistical analysis. | | | **Very Good**  -First study of rehabilitation intervention in this study group  - New knowledge about the fact that intervention was both safe and helpful with significant improvement in fatigue and other variables 12 month follow up. Important for clinical practice and further research | |
| **62**  Fusar Poli et al 2008 | | MFS:  All had verified diagnosis by Ghent 1 (all had dural ectasia) | | **Good**  -The design clearly described and reasoned.  -The design seems appropriate for the purpose.  -Statistical analyses with small sample | | **Good**  -Small sample size, but all had verified diagnosis.  -Presumably not representative for the MFS population in the country, but for people with MFS and durale ectasia  -Skewed gender distribution (75% female), age distribution good. | | **Acceptable**  -Compared to general population, but not on vitality | | **Good**  -Generic validated instrument (SF-36), reporting data on vitality.  -Discuss the instruments general validity.  -Nothing about suitability for the patient group. | | **Acceptable/good**  -Recruitment process, inclusion/exclusion described  **-**Missing data, response rate, non-respondents and drop-out analysis not described | | **Acceptable**  **-**Very short description of limitation of the study, no discussion. | | **Good**  **-**All verified diagnosis, appropriate study design and methods, but small sample size, use of standardized instruments and transparent description of recruitment process, limitation and pre/post statistical analysis  - Include a summary of pertinent literature | | | **Acceptable**  -Give some separate data on SF-36, vitality score, particularly for MFS patients with dural ectasia. Very little on fatigue and vitality. | |
| **52**  Ghanta et al 2015 | | MFS:  22 of 49 had verified diagnosis (Ghent 1 or genetic verified). Represent largest contemporary extent of MFS II TAAA repair. | | **Good**  -The study design is clearly described  -Design appropriate for the purpose when combining prospective and retrospective design.  -Statistical analyses with small sample. | | **Acceptable**  -Very small sample size, especially for the QoL part, and only half had verified diagnose.  -Presumably not representative for the MFS population in the country.  -Gender/age distribution good. | | **Good**  **-**Comparing with healthy controls matched for age/gender. Norm data for SF-36vt. | | **Acceptable**  - Generic validated instrument. (SF-12).  - Very limited description about validity of the instrument.  -Nothing about the suitability for the study groups | | **Good**  **-** Recruitment process inclusion/exclusion criteria process, respond rate described – limited description.  -Non-responders, missing data and drop-out analyses not described | | **Good**  -Good description of limitation, about: retrospective, small sample, possible bias, one center study.  -Discussed how to ameliorate this. | | **Acceptable**  -Only 13 of 24 had verified diagnosis, appropriate study design, very small study sample and challenges in statistical analysis not described. Transparent and comprehensive description of recruitment/selection process. | | | **Acceptable/good**  -First study assessing QoL and vitality/fatigue in MFS patients undergoing thoracoabdominal surgery  -Gives knowledge on this aspect | |
| **63**  Moon et al 2016 | | MFS:  All had verified diagnosis (Ghent 2). | | **Very good**  **-**The study design is clearly described and reasoned  -Appropriate for the purpose of the study and well described using an exploratory structural model.  -Adequate statistical analyses with a relatively huge sample size | | **Good**  -The sample size is relatively large, and all verified diagnosis  -Mostly including ”well-functioning participants”  -May represent a large sample of the population.  -Gender and age distribution not reported. | | **Good**  -Compared to results of general population (reference values) and other disease from published studies-but not on vitality/fatigue | | **Very good/good**  **-** Generic validated instrument for measuring fatigue (FSS) and separate data for SF-36 vitality score  -A comprehensive description of the instrument, the internal consistency, test/retest reliability and construct validity adequate for general population  -Mention: limitation not used disease specific measures | | **Good**  – Recruitment process, inclusion/ exclusion, response rate and non-respondents described.  -Missing data and drop-out analyses not described. | | **Very good/good**  - Comprehensive discussion of limitations: single center, the study sample (gender skewed, young, well-educated mild MFS), the measurement etc. Discussed how to ameliorate this. | | **Very Good**  -All verified diagnosis, appropriate study design, validated instruments and large sample size. Comprehensive and transparent description of the study process, recruitment and analysis.  -Thorough description of available knowledge prior to the study. | | | **Good/very good**  -Information about fatigue, and that fatigue is associated with decreased quality of life.  -First study using structural equation modeling to investigate quality of life in MFS  -Important results on how the different factors. like fatigue influence QoL  - | |
| **72**  Percheron et al 2007 | | MFS  All had verified diagnosis, (Ghent 1) | | **Good/Acceptable**  -Study design well described  -Design and methods seems appropriate for the purpose of the study  - Statistical analyses with very small sample size. | | **Acceptable**  -Low sample size, but all verified diagnosis  -Only including women  -Presumably not representative for the MFS population.  -Wide age-distribution. | | **Good**  Control group was 19 employees/  students (women) at hospital where the study was conducted, matched for age and anthropometric on fatigue. | | **Acceptable**  **-**Generic validated instrument for measuring fatigue.  - A short description of the instrument.  - Nothing about the suitability for the study group. | | **Good**  -Recruitment process, inclusion/exclusion, response rate, and non-respondents described.  -Missing data and drop-out analysis not described. | | **Good**  -Good description of limitations, particularly about the physical/ muscle measurement. | | **Good**  -All verified diagnosis, appropriate study design, but small sample size.  -Comprehensive description of measurement, statistical analysis, and limitations. | | | **Good**  Novel information, about fatigue and physical activity, greater fatigue in MFS, no differences in muscle fatigue.  -Give data on fatigue in MFS women | |
| **53**  Peters et al 2001 | | MFS  not verified diagnoses, only self-reported | | **Good**  -Study design is described and reasoned  - Design and methods seems appropriate for the purpose  -Statistical analyses with a relatively large sample  -Use of theoretical framework guiding the study | | **Acceptable**  -Relatively large study group, but not verified diagnosis.  -Presumably not representative for the MFS population in the country  -Gender distribution god, age distribution not reported. | | **Good**  -Compared with norm-data and other cardiac diseases from published data,  -No control groups | | **Acceptable**  -Use a generic illness perception instrument, including one question on prevalence of fatigue  -Discuss briefly the instruments general validity and reliability - Nothing about suitability for the patient group | | **Good**  - Recruitment process, inclusion/exclusion, response rate described  -Drop-out, non-respondent analyses and missing data not reported | | **Good/Very good**  Thorough information and discussion about limitation: study design, self-reported data, response rate, non-respondents, the study sample (self-selected, motivated). Discussed how this could be ameliorated  (separate section) | | **Good**  -Not verified diagnosis, appropriate study design and methods, large study sample, but very low response rate  -Thorough discussion about the study process and information about the instruments included, | | | **Good**  -Novel knowledge at the time the study was conducted.  -Extensive information on illness perception in MFS, also including some information about fatigue  -Some new knowledge of fatigue, prevalence and association and that fatigue was associated to concentration problems. | |
| **31**  Rand-Hendriksen et al 2007 | | All had verified MFS,  (Ghent 1) | | **Good**  -The design and method clearly described  -Appropriate for the purpose of the study  -Statistical analyses with very small sample size | | **Acceptable**  -Very small sample size, but all had verified diagnosis  -Presumably not representative for the MFS population in the country.  -Skewed distribution of gender (81% female), young age group. | | **Good**  -Compare to norm-data, published data on fatigue.  -No control group. | | **Good**  -Two generic standardized fatigue instruments (FSS/FQ)  -Description of the instruments, general validity and reliability  -Showed acceptable internal consistency  - Nothing about suitability for the patient group | | **Good**  -Recruitment process, inclusion/exclusion process response rate and non-response rate described  -Missing data and drop-out analyses not described. | | **Acceptable**  -Very limited description about limitation, one sentence about small number and few male (separate section) | | **Good**  -All verified diagnosis, appropriate study design, very small sample size and challenges related to statistical analyses with comprehensive testing.  -The study process is transparent described, but limitations not emphasized | | | **Very good**  -First study mainly focusing on fatigue in MFS  -Novel results, but not generalizable.  -Give extensive information on fatigue and correlations, to neurocognitive factors, although small sample | |
| **13**  Rand Hendriksen et al 2010 | | MFS:  All had verified MFS,  (Ghent 1) | | **Good**  - The design and method well-described and reasoned  -Design and methods seems appropriate for the purpose  -Adequate statistical analyses with relatively huge sample, including clinical examination | | **Good**  - Relative large sample size, and all verified diagnosis  -May represent a large amount of the patient population in the country  -Good distribution of gender and age. | | **Very good**  -Compare with healthy controls matched for age/gender from general population, and other studied of MFS and other patient groups (published data) on SF-36vt. | | **Good**  -Generic validated instrument (SF-36), reporting vitality data.  - Discuss the instrument general validity and reliability,  - Mention that the instrument may not be suitable for the study population | | **Good**  -Recruitment process, inclusion/exclusion, response rate and drop-out analysis were reported  -Non-respondents and missing data not reported. | | **Acceptable**  -Short description on potential limitations, mainly about the respondents (skewed to female) and instrument (may not be suitable for the study group)  (separate section) | | **Good**  -All verified diagnosis, appropriate study design, validated instruments, relatively large study sample  -Comprehensive and transparent description, but only short description of limitations. | | | **Acceptable**  -Present limited data on fatigue/vitality | |
| **64**  Rao et al 2016 | | MFS:  All had verified diagnosis confirmed by a genetic modified MFS criteria (Ghent 2) | | **Very good**  -The study design and methods well described, transparent and well founded.  -The study design seems very appropriate for the purpose  -Adequate statistical analysis | | **Very good**  –Relatively large sample size (n=230), all had verified diagnosis.  -May represent a large amount of the MFS population  -Good gender and age distribution | | **Good**  -Comparison with published norm-data, on SF-36vt  -No matched controls | | **Acceptable**  **-** Study specific question on fatigue, (not validated)  -Generic validated instrument (SF-36), reporting data on vitality.  -Description of the instrument, nothing about validity or reliability, or suitability for MFS  - Nothing about suitability for the patient group | | **Acceptable**  **-**Recruitment process, **i**nclusion/exclusion and response rate described  -Non-respondents, missing data and drop-out analysis not described.  -Data seems to be missing (different n in various sub-analyses) | | **Fair**  -Nothing about the study limitations. | | **Acceptable**  -All verified diagnosis, large study sample.  -Appropriate study design and methods, but some methodological questions (description of correlation between variables not examined in the result section)  -Limitations is not taken into account | | | **Good**  -Some information on fatigue contributing to the knowledge on fatigue in MFS  - Interesting and new findings compared to general population, but no discussion of possible explanations of their findings  - Results and discussion section not consistent | |
| **54**  Ratiue et al 2018 | | MFS:  not verified diagnosis, self-reported. | | **Good**  -Description of study design and methods described and transparent  -The study design seems appropriate  -Adequate statistical analysis, well described | | **Acceptable**  -Large sample size, but without verified diagnosis and very low response rate  -Presumably not representative for the MFS population in the country  **-**Skewed for gender (66% female), but good age-distribution. | | **Acceptable**  -No comparison with controls or norm data  -No control group  -No comparison to other population | | **Acceptable**  - Generic validated instrument (SF-36), reporting vitality.  -Nothing about validity, reliability  -Noting about the suitability for the patient group | | **Acceptable**  **-**Recruitment process, inclusion/exclusion and response rate described.  -Non-responders, missing data and drop-out analyses not described. | | **Very good**  -Comprehensive description of limitations: the response rate (2%), classification bias respondents bias, non-respondents etc. Attempted to minimize bias- and discussed how they tried ameliorated this bias. | | **Acceptable/good**  **-**Not verified diagnoses, very low response rate but large study sample.  -Appropriate study design and methods, validated instrument  **-**The study procedures, methods and analyses are transparent described, but limited description of the validity/reliability of the instrument used. | | | **Good/acceptable**  -Contribute to new knowledge  -Associations between mental capacity and QoL well described, but no comparison to other groups | |
| **65**  Schoormans et al 2012 | | MFS  All had verified diagnosis,  (Ghent 1) | | **Very good**  **-**The study design, methods and hypotheses thoroughly described.  -Study design seems very appropriate for exploring the hypotheses  - Very appropriate statistical analysis | | **Good**  -Relative large sample size and all had verified diagnosis.  **-**May represent a large part of the MFS population in the country.  -Good gender distribution, and age-distribution not described. | | **Good**  **-** Compare with healthy controls matched for age and gender, Dutch reference population, (published data), on SF-36,vt. | | **Good**  **-** Generic validated instrument (SF-36), reporting vitality.  -Discuss the instrument general validity and reliability  -Nothing about the suitability for the study group. | | **Good**  -Recruitment process, inclusion/exclusion response rate, non-respondents, drop-out analysis well described.  -Missing data not described. | | **Very good**  - Comprehensive discussion of limitations: the study sample (no cardiovascular MFS), small sample size, bias influence outcome and statistical challenge (use of Bonferroni correction). Discussed how these were ameliorated | | **Very good**  -All had verified diagnosis, relatively large study sample, appropriate study design and methods, validated instruments.  -Systematic, transparent, description of study process, and limitations are emphasized.  - Extensive comparison between gene-analyses and QoL | | | **Good**  -Gives new and more knowledge about gene- mutations and the association to fatigue.  -Although little information about fatigue | |
| **32**  Van Andel  et al 2022 | | MFS:  All had verified diagnosis (Ghent 2, for both groups). | | **Very good**  **-**The study design, methods comprehensively described and reasoned.  -The design and method very appropriate for the purpose of the study  -Statistical analyses seems very adequate. | | **Good**  -Relatively large sample size, and all verified diagnoses  -May be representative for MFS population in the country.  -Good gender and age distribution | | **Good**  -Compared to general population and subgroups, on fatigue  -No other diseases. or matches controls | | **Very good**  -Two generic validated instrument for measuring fatigue  -Thoroughly information and discuss validity/ reliability.  -Nothing about the suitability for the study group. | | **-Good**  - Recruitment process, inclusion/exclusion, response rate, drop- out and excluded respondents reported  -Nothing reported about missing data | | **Good/Very good**  **-**Comprehensive discussion about possible limitations: retrospective study design, self-reported medical information, measurements, lack of data etc. Discussed how these can be ameliorated  (separate section) | | **Good**  All verified diagnosis, relative large study sample, appropriate study design and methods, validated instruments  - Systematic and transparent description of methods, analysis and limitations.  -Comparing two groups with MFS- but the purpose not well described. | | | **Very good**  -Novel knowledge about fatigue.  -New information that losartan negatively associated with fatigue.  -Verifies that it is difficult to establish the causes of fatigue  - | |
| **55**  Van Dijk et al 2008 | | MFS:  Ghent 1 for those in the experimental group, but not mention for the rest | | **Good**  -The study deign, methods and research questions well described and defined  -The study design appropriate for purposes  - Statistical analysis with small sub-groups (experimental groups). | | **Acceptable**  -Small sample size (n=49), and verified diagnoses not described for all.  -Presumably not representative for the MFS population.  -Good gender and age distribution. | | **Good**  -Comparing with healthy Dutch population on all subscales of MFI-20, on fatigue  -Not matched controls, or other patient group | | **Good**  **-**Generic validated instrument (MFI-20),  -Discuss the general validity and reliability,  -Nothing about the suitability for the patient group. | | **Acceptable**  **-**-Recruitment process, inclusion/exclusion and response rate described.  -Missing data, non-respondents and drop-out analysis not described. | | **Very good**  -Comprehensive description and discussion about limitation: The study sample, not matched controls etc. Thoroughly discuss how these were ameliorated  (separate section) | | **Good**  -Only few had verified diagnosis, and small study sample, but appropriate study design and methods, validated instruments.  -Systematic and transparent description of the study process and limitations. . | | | **Good**  -New and novel knowledge about fatigue  -Good interpretation of fatigue and possible associations.  -New information about associations between fatigue and orthostatic intolerance. | |
| **73**  Vanem et al 2021 | | MFS:  All had verified diagnosis,  Ghent 1 | | **Very Good**  -The study design, methods and research questions well described and defined  -The study design seems very appropriate for the purpose  - Statistical analyses based on relatively small sample size, but probably it constitute a large amount of the MFS local population | | **Good**  -Small sample but all with verified diagnosis  -May represent longitudinal understanding of MFS population.  -Skewed distribution of gender and age. | | **Very good**  -Comparing both with healthy controls, longitudinal and other patient groups, | | **Good**  -Generic validated instrument (SF-36), reporting data on vitality.  -General validity and reliability discussed  -Nothing about suitability for the patient group. | | **Very good**  - Recruitment process,  inclusion/exclusion, respond rate, non-respondents, missing data and drop-out thoroughly described and handled  - | | **Very Good/Good**  -Comprehensive description of limitations; small study cohort, lack of relevant variables (socioeconomic) etc. Discussion on how these potential biases could be ameliorated. | | **Very good**  -All verified diagnoses, small study sample, appropriate study design, methods and validated instruments  - Systematic and transparent description of the longitudinal study process  -Extensive comparison between clinical examination on diagnostic features and QoL | | | **Acceptable**  Little knowledge about vitality and fatigue, but first longitudinal study of QoL in a sample with verified diagnoses.  Finding stability vitality scores over 10 years | |
| **66**  Velvin et al 2015 | | MFS  All had verified diagnosis, (Ghent 1) | | **Good**  -Study design is clearly described  -The design and method seems reasonable for the purpose  -Statistical analyses is appropriate, with a relatively small sample. | | **Good**  -Relatively small sample size, but all with verified diagnosis.  -May represent at large population of MFS in the country  -Good gender and age distribution. | | **Good**  -Comparing with norm data and other patient groups (published data) - Not on fatigue.  - No matched control groups  . | | **Good**  -Generic validated instrument (FSS).  -Describe and discuss the general validity, reliability, nothing about the suitability for the study group  -Present cut-off values for severe fatigue  -Nothing about suitability for the patient group | | **Acceptable**  -Recruitment process, inclusion/exclusion response rate and non-respond analysis described  -Missing data and drop-out analysis not described. | | **Fair**  -Limitations purely described and not discussed | | **Acceptable**  -All verified diagnosis, small study sample, appropriate study design, methods and validated instruments  - Transparent description of the study process, but nothing about limitations. | | | **Acceptable**  -New knowledge about how fatigue may be associated with work participation, but little about how it influence work participation. | |
| **67**  Velvin et al 2016 | | MFS  All had verified diagnosis, (Ghent 1) | | **Good**  - Study design clearly described  -The study design seems appropriate for purpose  -Statistical analyses is appropriate with a relatively small sample | | **Good**  **-**Relatively small sample size, but all with verified diagnosis  -May represent at large population of MFS in the country  -Good gender and age distribution. | | **Good**  -Comparing with norm data, and other patient groups (published data) - Not on fatigue.  -No matched controls | | **Good**  **-**Generic validated instrument of fatigue (FSS).  -Discuss general validity and reliability  -Nothing about suitability for the study group | | **Good**  - Recruitment process, inclusion/exclusion,  response rate, non-respondents and missing data described  -Drop-out analysis not reported. | | **Good**  -Comprehensive description of limitations: Response rate, not matched controls, self-constructed questionnaire, small sample size influence on statistical power.  Discussed how they were tried ameliorated (separate section) | | G**ood**  -All verified diagnoses, small sample, appropriate study design, methods and validated instrument.  -Transparent description of the study process and limitations. | | | **Good**  -First study investigating associations between Satisfaction with life and fatigue. How fatigue may influence life satisfaction. | |
| **Ehlers Danlos syndrome** | | | | | | | | | | | | | | | | | | | | |
| **68**  Schubart et al 2019 | | Heritable Connective tissue phenotypes (HCTP)  Verified diagnosis criteria 2015 | | **Acceptable**  -The study design described  -Design and method seems appropriate according to the purpose of the study  -Statistical advanced analyses on small subgroups (phenotypic clusters) | | **Good**  -A relatively large sample of EDS with verified diagnoses, but with small subgroups  -Presumably not representative for the HCTP population in the country  -Skewed gender distribution, age-distribution not described. | | **Good**  -Comparing between subgroups between those with High Symptom Burden Subgroup and those without.  -Not with other diseases, general population or matched controls | | **Good**  -Several validated standardized instruments were used to measure fatigue and other aspect  -No discussion about general validation or reliability or suitability for the study group | | **Good**  -Recruitment process, inclusion/exclusion, missing data, response rate thoroughly described  -Nothing about non-respondent or drop-out analysis | | **Good**  -Comprehensive description of limitation: diagnostic criteria (2017 nosology), cluster analysis, statistical imputation, cluster algorithm etc. Discussed how they were tried ameliorated. | | **Good**  -All verified diagnosis, relative large study sample, appropriate design, method sand validated instrument.  -Transparent description of the study process, inclusion, selection, analyses and limitations. | | | **Acceptable**  -Little new information about fatigue on the different subgroups of EDS (HCTP)  -Shows some differences in mental fatigue between patients with vEDS and other EDS types.  -Has very limited data on fatigue in vEDS. | |
| **57**  Voermans et al 2010 | | EDS:  Included several subgroups of EDS, without medically verified diagnoses | | **Good**  -The study design and methodology well described  -Design and methods seems appropriated for examine the purpose  -Statistical analyses of subgroup on/between very small subgroups | | **Acceptable**  -Relatively large total study sample, but small subgroups, all with self-reported diagnosis  -Probably not representative for the population  -Skewed gender distribution, but good age-distribution | | **Good**  -Comparing prevalence of fatigue between different subgroup of EDS  -Not with general population or other patient groups  -Not matched controls | | **Good/Acceptable**  -Generic standardized fatigue instrument (CIS).  -No information on general validity or reliability  -Nothing about suitability for the study sample | | **Good**  -Recruitment process, inclusion/exclusion, response rate and missing data described  -Noting about non-respondents or drop-out analysis | | **Good**  -The limitations described: study cohort (recruited from MFS foundation),, lack of non-respondent analysis  -Discussed how they were ameliorated | | **Good**  -Many respondents without verified diagnosis, but large study sample, but small subgroups.  -Appropriate study design, method and validated instruments  -Extensive analyses on possible associations to fatigue in EDS, although not specified on EDS subtypes | | | **Good**  -One of few studies reporting fatigue in vEDS  -New information about fatigue and EDS, in different subgroups. In addition what characterizes those with severe fatigue vs those with non-severe fatigue. | |
| **Marfan syndrome and Ehlers Danlos syndrome** | | | | | | | | | | | | | | | | | | | | |
| **56**  Verbraecken et al 2001 | | MFS/EDS:.  Self-reported diagnosis  (report of their previously diagnosis) | | **Good**  -The design is well descried  -The study design and methodology seems appropriate for the purpose  -Statistical analyses limited described and relatively advanced analyses for small sample size | | **Acceptable**  -Small total study sample with self-reported diagnosis, and very small subgroups  -Probably not representative for the study population.  -Good gender distribution and age-distribution nor reported. | | **Good**  -Compared with 24 healthy controls (co-workers at the institution),  -Controls matches on age, gender and BMI matched  -Not with other diseases. | | **Good**  -Generic standardized instrument for QoL (SF-36, vitality score).  -Discuss the instrument general validity and reliability.  -Nothing about suitability for the study group | | **Acceptable**  **-**Recruitment process, inclusion/ exclusion well described  -Nothing about response rate, missing data, non-respondents or drop-out analysis.  , | | **Good**  -Comprehensive description of limitations: study cohort, (from MFS foundation, gender skewed, small sample on sub-groups), study design.  -Discussed how they were tried ameliorated | | **Good**  -Not verified diagnoses, small study sample (and sub-group analysis)  -Appropriate study design, method and validated instruments  -Transparent description of the study process, but limited description of using advanced statistical analyses on small groups, but limitations well described  -Pilot study of new issues. | | | **Acceptable**  - Present some data on vitality and daytime sleepiness in a small group of MFS | |
| **Vascular Ehlers Danlos (vEDS) Syndrome and Loeys Dietz syndrome (LDS).** | | | | | | | | | | | | | | | | | | | | |
| **11**  Johansen et al 2019 | | LDS/vEDS:  All medically genetic verified diagnosis | | **Good**  -Study design and method well described and reasoned.  -The design seems appropriated for the purpose  -Adequate statistical analyses, but relatively small sample. | | **Good**  -Relatively small study sample, but all had molecularly verified diagnoses  -Probably deals with a large proportion of the LDS and vEDS national population  -Good gender- and age distribution | | **Acceptable**  -Comparing physical activity with general population- not on fatigue,  -No matched controls | | **Good**  -Using a standardized fatigue instrument  -Information about the instrument general validity and reliability.  -Nothing about the suitability for the study population | | **Good**  Recruitment process, inclusion/exclusion, response rate, non-respondents described.  -Drop-out analysis and missing data not reported. | | **Good**  -Comprehensive description of limitations; study sample (small subgroups), statistical power analysis (very small subgroups). -Discussed how they were tried ameliorated.  (separate section) | | **Good**  -All verified diagnosis, but small study sample (very small sub-group), appropriate study design, methods and validated instruments.  -Extensive analyses on possible associations between physical activity and other factors- including fatigue | | | **Good**  -Find new information about fatigue negative associated to physical activity  -Have data on mean fatigue score and associations to physical activity in patients with verified diagnoses | |
| **69**  Johansen et al 2021 | | LDS/vEDS:  All medically genetic verified diagnosis | | **Good**  **-** Study design and method well described and reasoned  -The design seems appropriated for the purpose  -Adequate statistical analyses, but relatively small sample  . | | **Good**  -Relatively small study sample, but all had molecularly verified diagnoses  -Probably deals with a large proportion of the LDS and vEDS national population  -Good gender- and age distribution | | **Good**  -Comparing to general population and other patient groups (published data) - not on fatigue.  -Not matched controls | | **Good**  -Using a standardized fatigue instrument  -Information about the instrument general validity and reliability  -Nothing about the suitability for the study population | | **Good**  -Recruitment process, inclusion/exclusion, response rate, non-respondents described.  -Drop-out analysis and missing data not reported | | **Good**  Comprehensive description of limitations; study sample (small subgroups), statistical power analysis (very small subgroups). -Discussed how they were tried ameliorated.  (separate section) | | **Good**  - All verified diagnosis, but small study sample (very small sub-group), appropriate study design, methods and validated instruments  -Extensive analyses on possible associations between Life satisfaction and other factors- including fatigue  . | | | **Good**  -Study with description of quality of life in these patient groups, and associations to fatigue.  -Have data on mean fatigue score and associations Life Satisfaction | |
| **16**  Johansen et al 2022 | | LDS/vEDS:  All medically genetic verified diagnosis | | **Good**  **-** Study design and method well described and reasoned  -The design seems appropriated for the purpose  -Adequate statistical analyses, but relatively small sample | | **Good**  -Relatively small study sample, but all had molecularly verified diagnoses  -Probably deals with a large proportion of the LDS and vEDS national population  -Good gender- and age distribution | | **Good**  -Fatigue compared to published data on general population and other patient group, and between LDS and vEDS  -Not matched controls | | **Good**  -Using a standardized fatigue instrument  -Information about the instrument general validity and reliability  -Nothing about the suitability for the study population | | **Good**  -Recruitment process, inclusion/exclusion, response rate, non-respondents described  -Drop-out analysis and missing data not reported | | **Good**  -Comprehensive description of limitations; study sample (small subgroups), statistical power analysis (very small subgroups)  -Discussed how they were tried ameliorated  (separate section) | | **Good**  - All verified diagnosis, but small study sample (very small sub-group), appropriate study design, methods and validated instruments  -Extensive analyses on possible associations between fatigue and other factors | | | **Very Good**  -The first study to investigate prevalence of fatigue and chronic pain associations to clinical and demographic factors in patients with verified diagnosis | |
| **sHTAD (including LDS, MFS and other sHTADs)** | | | | | | | | | | | | | | | | | | | | |
| **58**  Thijssen et al 2020  Male and female differences | | sHTADs:  86,5 % verified MFS, (Ghent 2).  No description of the diagnoses to the rest | | **Good**  -Study design well-descried  -Design appropriate for the purpose  -Adequate statistical analysis, but the description of the analysis is very limited | | **Good**  -Relative large total sample size and the majority had verified diagnosis, but small subgroups.  -Probably not be representative for the study population  -Good distribution of gender, age-distribution not described | | **Good**  -Comparing between men and women, and with general population, not other patient groups  **-** No matched controls | | **Good**  -Using a generic standardized instrument for QoL (vitality score).  -Discuss the instrument`s validity and reliability  -Nothing about the suitability to the study group | | **Good**  -Recruitment process, inclusion/exclusion, dropout, non-respondents thoroughly described  -Response rate or missing data not described. | | **Good**  Comprehensive description of limitations: drop-out, non-respondents, the representativeness of the study.  Discussed how they were tried ameliorated (separate section) | | **Good**  -Nearly all verified diagnosis, relative large study sample, but analysis of small subgroups, appropriate study design, methods and validated instruments.  -Transparent description of the study process, analyses and limitations. | | | **Good**  -Present data on male/female differences in vitality and fatigue symptoms, including a relatively large group of patients | |
| **Children** | | | | | | | | | | | | | | | | | | | | |
|  | |  | |  | |  | |  | |  | |  | |  | |  | | |  | |
| **70**  Warnink-Kavelaars et al 2020 | | MFS:  All children had verified diagnosis, Ghent 2 | | **Good**  -The design and method well- described and well-founded  -The study design seems appropriate for purpose  -The study design and process are described and well-founded  -Adequate statistical analysis. | | **Good**  -The sample size is relatively large, and all children had verified diagnosis  - May be representative for the study population in the country  -Goode gender and age distribution | | **Good**  -Comparing with norm data, on fatigue  -Comparing between mothers and fathers  -Not matched controls. | | **Good**  -Validated screening instrument for measuring clinical distress, everyday problems including fatigue.  -Describe validity, reliability.  constancy was mentioned.  -Nothing about suitable tor the study group. | | **Acceptable/Good**  -Recruitment process, inclusion/ exclusion well described.  -Response rate, non-respondent, missing data and drop-out not described. | | **Good**  Comprehensive description of limitations: Study sample size, recruitment, measurement methods.  -Discussed how they were tried ameliorated. | | **Good**  -All verified diagnosis, relative large study sample, appropriate study design, method and validated screening instruments.  -Systematic and transparent description the study process and recruitment process. | | | **Good**  -Some description of the prevalence of fatigue in children with MFS, and description of prevalence fatigue in their parents to children with MFS. | |
| 61  Warnink-Kavelaars, et al 2021 | | HCTD (MFS, LDS, EDS and hEDS):  Unclear if the children had verified diagnosis, not described (parents verified) | | **Good**  -The design and method well- described and well-founded  -The study design seems appropriate for purpose  -The study design and process are described and well-founded  -Adequate statistical analysis, but small subgroup analysis | | **Acceptable**  -The sample size is relatively large, but small subgroups and verified diagnose not described  -Probably not representative for the study population  -Good gender- and age distribution | | **Good**  -Calculated T-score based on norm values for PROMIS, and compared to the norm population, on fatigue, and sub-groups.  -No matched controls or other diseases | | **Good**  -Generic standardized instrument- PROMIS  -Discuss the instrument`s general validity and reliability  -Nothing about suitable for the study group | | **Good**  -Recruitment process, inclusion/exclusion, missing data, response rate well described.  -Non-respondents and drop-out not described | | **Good**  -Description of limitations study cohort (small sample, parents reported diagnosis and appropriate study design  - Discussed how they were tried ameliorated | | **Good**  -Unclear verified diagnosis, relatively large sample size, but small subgroups, but very appropriate study design, methods and validated instruments  -Comprehensive and transparent description of the study process | | | **Very good**  **-**The first study with to investigate disability and fatigue in children with HCTD with validated instruments  New and interesting findings | |
| **Quality assessment of qualitative studies** | | | | | | | | | | | | | | | | | | |  |  |
| **Quality assessment criteria**   1. Is the research questions(s) clearly and explicitly stated? 2. How was the participants selected (described selection process)? 3. The researchers role and has it been taken in account? 4. Is the method appropriate for collecting data? 5. Is the method appropriate for analyzing the data and for ensuring scientific rigor? 6. The credibility of the study ( as a whole) 7. Do the study contribute to novel knowledge on the particular issue (fatigue)? | | | | | | | | | | Ratings: Very good, Good, Acceptable, Fair and Poor | | | | | | | | |  |  |
| **Author, year** | | **Diagnosis(es)** | | **Research question(s)** 1 | | **Recruitment**2 | | **The role of researcher(s)**3 | | **Appropriate method**4 | | **Appropriate analysis(es)**5 | | **Limitations**6 | | **Credibility**7 | **Contribute to new knowledge**8 | |  |  |
| **Adults** | | | | | | | | | | | | | | | | | | |  |  |
| **12**  Velvin et al 2021 | | MFS, LDS, vEDS.  All verified diagnoses | | **Good**  -The aim/research questions are clearly formulated  -Introduction highlight why research is necessary  -Qualitative design seems suitable to investigate the patients perspectives and experiences | | **Good**  -Patient recruitment and sampling process well described | | **Good**  -The role of the researchers have been discussed both in the data gathering and the analytic process, however not in detail | | **Good**  -Method seems suitable for data collection on the research questions  -Procedures for interviews, and analyses described | | **Good**  -Analytic methods described and seems appropriate, and thoroughly described | | **Good**  -Good description of limitation: retrospective perspective implies bias, study cohort, concept, methodology and researchers role  -Discuss how they were tried ameliorated  (separate section) | | **Good**  -Transparent description about methods, and theoretical background, limitation and study process.  -All verified diagnosis  - Fatigue was secondary outcomes | **Acceptable**  -Little new information about fatigue, but something on how fatigue impact physical activity. | |  |  |
| **Adolescent/children** | | | | | | | | | | | | | |  | |  |  | |  |  |
| **59**  Kelleher et al 2015 | | MFS:  Not verified diagnoses, only self-reported | | **Good**  -The aim/research questions are clearly described  -Introduction highlight why research on the topic is needed  -Qualitative design with Facebook posts may be suitable for answer the research questions | | **Acceptable**  -Data selection process is well described, this form of data gathering renders no control over recruitment  -However, there is an uncertainty to post without verified diagnosis | | **Acceptable**  -The researchers role was not described or taken into account | | **Very good**  -Method seems very appropriate for the purpose  -Procedures for data gathering and coding thoroughly described | | **Good**  -Only descriptive analyses with numbers and percentages, and some quotes. | | **Good**  -Good description of limitations: pilot study, small sample, lack of information about respondents  -Discussion on how they could be ameliorated | | **Good**  **-**Transparent description of the study process  -Selected range of internet platforms  -Not verified diagnosis | **Good**  -New and innovative knowledge about fatigue Indicating that fatigue is not very emphasized among youth with MFS on social media. | |  |  |
| **74**  Warnink-Kavelaars et al 2019 | | MFS:  All had confirmed diagnosis (pathogenic FBV1) | | **Very good**  -The research questions were clearly and explicitly stated. (two main research questions based on previous research/literature)  -Qualitative design seems suitable to answer the research questions in the target group. | | **Good**  -The selection process described and participants recruited from clinic (may be skewed sample)  -All verified diagnoses | | **Acceptable**  -Nothing about the researchers role and if this has been taken into account? | | **Very good**  -The method seems very appropriate for collecting data.  --Rationale for interview guide and procedures for data gathering and coding very well described | | **Very good**  -The method (thematic analyses) for analyzing data was very appropriate and the authors explicitly described strategies for ensuring trustworthiness and credibility – by investigator triangulation for ensuring rigor. | | **Very good**  Comprehensive description of limitations: recruitment, study sample (Dutch) cultural differences etc.  Discussion on how they could be ameliorated. | | **Very good**  -The credibility of the study very seems good.  -Transparent description of study process  -Including youth with verified diagnose. | **Very good**  -First qualitative study investigating fatigue in adolescent with MFS, but limited results on how fatigue may influence life-situations. | |  |  |
| **60**  Warnink-Kavelaars, et al 2019a | | MFS:  No description that the children had verified diagnosis. | | **Good**  -Aim/research question are clearly formulated  -Introduction highlight why research on this topic is necessary  -Qualitative design seems appropriate for answering the research questions. | | **Good**  -Selection well-described purposively for diversity of the sample.  -Although the diagnosis is not described, recruitment through a MFS expert centre indicate that the target group has MFS | | **Acceptable**  -The researchers’ role has not been described or taken in account? | | **Very good**  -The method seems very appropriate for the purpose, combining focus groups with individual interviews  -Rationale for interview guide and focus groups, procedures for data, gathering and coding well-described | | **Good**  Comprehensive description of analyzing method (thematic analyses) both for individual and focus groups,  -Also describing strategies for ensuring trustworthiness and credibility- by investigator triangulation to ensure scientific rigor.  Methods for enhancing trustworthiness of analyses well described | | **Very good**  Comprehensive description of limitations: recruitment, study sample, subjective parental observation, cultural differences etc.  Discussion on how they could be ameliorated. | | **Good**  -Overall, the credibility seems good.  -Transparent description of study process  -Give a credible description of parents opinion of how MFS impact their child.  -Nor described verified diagnosis, and fatigue was secondary outcome. | **Good**  -One of few studies that qualitatively explore parents experiences and report how MFS symptoms, including fatigue, tiredness affects the child | |  |  |

**Qualitative assessment of review articles**

| Quality assessment criteria  1. Is the review question clearly and explicitly stated?  2. Were the inclusion criteria appropriate for the review question?  3. Was the search strategy appropriate?  4. Were the criteria for appraising studies appropriate?  5. Was critical appraisal conducted by two or more reviewers independently?  6. Were there methods to minimize errors in data extraction?  7. Were the methods used to combine studies appropriate?  8. How is the credibility of the study? (limitations described, transparency, method, analyses and total impression)  9. Contribution to new knowledge? (benefits worth the harms and costs), implication for practice and recommendation for further research. | | | | | | Ratings: Very good, Good, Acceptable, Fair and Poor | | | | |
| --- | --- | --- | --- | --- | --- | --- | --- | --- | --- | --- |
|  |  |  |  |  |  |  |  |  |  |  |
| **Authors** | **Diagnosis(es)** | **Research questions**1 | **Inclusion criteria**2 | **Search strategy**3 | **Criteria for appraisal**4 | **Process of critical appraisal**5 | **Methods for minimizing error I data extraction**6 | **Methods for combining studies**7 | **Credibility**8 | **Contribution to new knowledge of fatigue**9 |
| **20**  Nielsen et al 2019 | MFS  All types of articles on MFS | **Good**  -The review questions were clearly and explicitly stated. | **Good**  -Well described, both the inclusion and exclusion criteria, and appropriate for the research questions. | **Good**  -The search strategy was systematic and well described, and appropriate for the research questions. | **Not relevant**  -No criteria for appraisal of the quality of included studies. | **Good**  -Not relevant, no quality assessment of included articles were conducted. | **Acceptable**  -Limited described. | **Good**  -Used thematic analyses to combine data from heterogeneity studies. | **Good**  -Thorough description of limitations.  -Detailed thematic presentation of findings on fatigue, across studies, however lack of critical appraisal limits the credibility. | **Good**  -Give an updated review on available studies on psychosocial aspects of MFS, including thematic analyses of fatigue |
| **19**  Velvin et al 2014 | MFS  All types of articles on MFS | **Good**  -The review questions were clearly and explicitly stated | **Good**  -Both inclusion/ exclusion criteria well described | **Good**  -The search strategy and search words was systematic and well described, and a | **Good**  -Using validated criteria for quality assessment, revised for rare diseases | **Good**  -Used validates criteria used for assessment of included articles r | **Acceptable**  Described -blinding and discussion of disagreements between two or several authors | **Good**  -Used thematic analyses to synthesize the results from included articles | **Good**  -Thorough description of limitations. | **Acceptable**  -Little information about fatigue |
| **51**  Velvin et al 2019 | sHTAD  No articles on other sHTAD were found | **Good**  -The review questions were clearly and explicitly stated | **Good**  -Both inclusion/ exclusion criteria well described | **Good**  -The search strategy and search words was systematic and well described, and a | **Good**  -Using validated criteria for quality assessment, revised for rare diseases | **Good**  -Used validates criteria used for assessment of included articles r | **Acceptable**  -Described -blinding and discussion of disagreements between two or several authors | **Good**  -Used thematic analyses to synthesize the results from included articles | **Good**  -Thorough description of limitations. | **Acceptable**  -Little information about vitality or fatigue |
