# Supplementary material for: Fatigue in patients with syndromic heritable thoracic aortic disease: a systematic review of the literature and a qualitative study of patients’ experiences and perceptions
Source: Orphanet J Rare Dis. 2023 May 19;18:119. doi: 10.1186/s13023-023-02709-2 (PMC10199502; doi:10.1186/s13023-023-02709-2)
Supplement: Supplementary file 4 — Additional file 4. Steps of the inductive systematic text analysis [file 13023_2023_2709_MOESM4_ESM.docx]

**Supplementary file 4. Steps of the inductive systematic text analysis**

| **Steps of STC** | **Means of establishing trustworthiness/robustness** |
| --- | --- |
| **Step 1: Overview**  Independently conducted by the researchers (GV/HJ)  **Step 2: Coding**  Independently by 2 researchers (GV/HJ)  **Step 3: Condensation:**  First independently conducted by 2 researchers (GV/HJ), then co-authors (TB/AØG).  **Step 4: Synthesizing**  First independently by 2 researchers (GV/HJ), then by co-authors (TB/AØG). | **-**Achieving an overview of the data material, by reading the text several times  -Bracket our preconceptions with sharp awareness for the participants voice  -Looking for preliminary themes  -The two researchers discussed tentative themes  -Systematic reviewing the text line by line to identify meaning units  -Use of preliminary themes framework and tentative themes  -Identify and organize data elements according to research questions  -Coding-identifying, classifying and sorting meaning units into categories and themes  -The categories were sorted, abstracted and condensed into themes with internal homogeneity  -The themes and categories were discussed among the research team to achieve consensus  -Reconceptualising – researchers putting pieces of text together again  -Present analytic text with most salient content and meaning  -Analytic distance and discussions  The underlying meaning in the themes and categories were linked together to create the main themes. |
